# *In vitro* anti-HIV and cytotoxic effects of pure compounds isolated from *Croton macrostachyus* Hochst. Ex Delile

**DOI:** 10.1186/s12906-022-03638-6

**Published:** 2022-06-15

**Authors:** Ermias Mergia Terefe, Faith A. Okalebo, Solomon Derese, Moses K. Langat, Eduard Mas-Claret, Nada H. Aljarba, Saad Alkahtani, Gaber El-Saber Batiha, Arabinda Ghosh, Eman A. El-Masry, Joseph Muriuki

**Affiliations:** 1 Department of Pharmacology and Pharmacognosy, School of Pharmacy and Health Sciences, United States International University-Africa, P.O. BOX 14634 – 00800, Nairobi, Kenya; 2 Department of Pharmacology and Pharmacognosy, College of Health Sciences, University of Nairobi, Nairobi, Kenya; 3 Department of Chemistry, University of Nairobi, Nairobi, Kenya; 4 Royal Botanic Gardens, Kew, Kew Green, Richmond, Surrey, TW9 3AE UK; 5 Department of Biology, College of Science, Princess Nourah bint Abdulrahman University, P. O. Box 84428, Riyadh, 11671 Saudi Arabia; 6 Department of Zoology, College of Science, King Saud University, P. O. Box 2455, Riyadh, 11451 Saudi Arabia; 7 Department of Pharmacology and Therapeutics, Faculty of Veterinary Medicine, Damanhour University, Damanhour, AlBeheira 22511 Egypt; 8 Microbiology Division, Department of Botany, Gauhati University, Guwahati, Assam 781014 India; 9 Microbiology and Immunology unit, Department of Pathology, College of Medicine, Jouf University, Sakaka, Al-Jouf Saudi Arabia; 10 Department of Medical Microbiology and Immunology, College of Medicine, Menoufia University, Shebin El Koum, Egypt; 11 Centre for Virus Research, Kenya Medical Research Institute, Nairobi, Kenya

**Keywords:** *Croton macrostachyus*, HIV-1, Anti-HIV, Lupenone, Lupeol acetate, Betulin and cytotoxicity effects

## Abstract

*Croton macrostachyus* is an important plant in traditional African medicine, widely utilized to treat a variety of diseases. In Kenya, HIV-infected patients use leaf and root decoctions of the plant as a cure for cough, back pain, bleeding, skin diseases, warts, pneumonia, and wounds. This study aimed to evaluate the anti-HIV activities and cytotoxic effects of extracts and chemical constituents isolated from *C. macrostachyus.* In our previous study we demonstrated that the hexane, CH_2_Cl_2_, ethyl acetate and methanol soluble fractions of a 1:1 *v/v/* CH_2_Cl_2_/MeOH crude extracts of the leaves and stem bark of *C. macrostachyus* exhibited potent anti-HIV activities against HIV-1 with IC_50_ values ranging from 0.02–8.1 μg/mL and cytotoxicity effects against MT-4 cells ranging from IC_50_ = 0.58–174 μg/mL*.* Hence, hexane soluble extract of 1:1 *v/v/* CH_2_Cl_2_/MeOH crude extract of the leaves of *C. macrostachyus,* that was more potent against HIV-1 at IC_50_ = 0.02 μg/mL was subjected to column chromatography leading to the isolation of 2-methoxy benzyl benzoate (1), lupenone (2), lupeol acetate (3), betulin (4), lupeol (5), sitosterol (6) and stigmasterol (7). Lupenone (2), lupeol acetate (3) and betulin (4) exhibited anti-HIV-1 inhibition at IC_50_ = 4.7 nM, 4.3 and 4.5 μg/mL respectively. The results obtained from this study support the potential of *C. macrostachyus,* as a source of anti-HIV constituents.

**Supplementary Information:**

The online version contains supplementary material available at 10.1186/s12906-022-03638-6.

## Introduction

Since the beginning of the pandemic caused by the human immunodeficiency virus (HIV), more than 75 million people all over the world have become infected with the virus [1]. HIV remains a significant threat to public health around the world, as evidenced by the fact that it has resulted in the loss of 36.3 million lives [1]. The number of people that were infected with the virus in 2019 was close to 2 million [2]. More than 300,000 people died as a result of the outbreak in southern and eastern Africa in the same year that the global death toll caused by the virus reached more than half a million. There were 680,000 deaths attributed to HIV-related causes in the year 2020, while there were also 1.5 million new HIV infections. According to data collected from around the world, the number of individuals living with HIV is expected to reach 37.7 million by the end of the year 2020. More than two thirds of these people (25.4 million) reside in the WHO African Region [1].

After the discovery of zidovudine in 1987 [3, 4] there have been significant developments in the field of antiretroviral therapy (ART). Zidovudine was given as a monotherapy to patients with advanced, symptomatic disease at a dose of five times a day at that time. It wasn’t until the middle of 1996 that researchers realized that using three or more antiretroviral medications at the same time had significantly better results [4]. This combination therapy (comprising at least three ARV agents) has shown a significant decline in viral replication and improvement in patients’ quality of life [5]. Although ART has contributed significantly to reducing death due to HIV, toxicities of the drugs, long-term effects due to lifelong therapy, and drug resistance are challenging problems for the success of HIV care and treatment [6]. HIV/AIDS has a detrimental economic, psychosocial, and health-related impact on countries, households, and individuals around the world. As the number of people infected with HIV rises, medical treatment costs rise, and national resources shift to the HIV/AIDS program [7].

Furthermore, because HIV-infected individuals are susceptible to a variety of opportunistic infections (OI), which can reduce their productivity and shorten their lives, co-administration of other drugs to treat OI might result in drug interactions that can impair ARV therapy or induce severe side effects [8]. The development of drug resistance has also posed a great challenge for treating clients since many cases show that patients do not respond, even to second-line regimens. Moreover, this problem continues to be a danger to the worldwide struggle to end the epidemic by 2030 [9]. Hence, there is a need for the discovery of novel drugs. One of the systematic approaches to tackle these challenges is to discover new drugs by identifying bioactive antiretroviral compounds from natural products. Currently, *Croton* species have gained attention in providing different bioactive phytochemicals used to treat bacterial and viral infections [10], and many *Croton* plants are used in ethnomedicine. This study evaluated the antiretroviral activity of *C. macrostachyus* using *in vitro* approach.

*C. macrostachyus* is a medium-sized tall tree or shrub that grows up to 30 mt [11, 12]. It is commonly known as a “broad-leaved *Croton*” or “rush foil.” It has various local/vernacular names- in Africa, including “*bisana*” in Ethiopia, “*msinduzi, mutundu*” in Kenya [12–17]. *C. macrostachyus* has a wide distribution in Africa, wherein in Kenya, it is found in the Karura Forest and many areas with significant rainfall [18]. *C. macrostachyus* is used as a remedy for a variety of illnesses. The plant possesses various medicinal properties [19] and treats constipation in Ethiopia, Cameroon, Rwanda, Kenya, Tanzania, Somalia, and Uganda. Usually, the decoction, macerated leaf, stem bark, or root is used [20–22]. In Kenya, leaf and root decoction is used by many patients, including HIV-infected patients, as a cure for cough, back pain, bleeding, skin diseases, warts, pneumonia, and wounds [22–26]. In Kenya, *C. macrostachyus* bark juice, leaf, and root decoction are used as a remedy for backache, bleeding, cancer, colds, cough, diarrhea, dysmenorrhoea, east coast fever, malaria, measles, obesity, pneumonia, ringworm, skin diseases, typhoid, warts, and wounds [27–29]. The leaves of *C. macrostachyus* are used by farmers in Kenya as biological pest control when mixed with tobacco (*Nicotiana tobacuum* L.) and boiled overnight [11]. In addition, roots of *Cucumis ficifolius* are often used in combination with *C. macrostachyus* bark as a remedy for abdominal and stomach pain [30]. Similarly, *Allium sativum* is given with *C. macrostachyus* to treat malaria [28, 29].

Antibacterial effects of *C. macrostachyus* against *N. gonorrhea* [31], *B. cereus*, *E. coli* and *P. aeruginosa* [32]*,* and *S. pyogenes* [33] have been reported. In a related study, Obey *et al.,* [25] reported that the ethyl acetate extract of stem bark of *C. macrostachyus* has good antibacterial activity against *E. coli*, *S. typhi*, *K. pneumoniae*, *E. aerogenes*, and *L. monocytogenes.* Taye *et al.,* [33] demonstrated the antibacterial activity of methanol leaf extract against *Streptococcus pyogenes* with a minimum bacterial concentration (MBC) value of 7.81 mg/mL. The antimycobacterial activity of *C. macrostachyus* in an i*n vitro* experimental study was reported by [34]. Semenya and Maroyi [35], also demonstrated the antimycobacterial activity of methanolic leaf extracts of *C. macrostachyus* with minimum inhibitory concentration (MIC) values ranging from 12.5 to 100 𝜇g/mL. This study demonstrated that *C. macrostachyus* has potential as an herbal medicine in the treatment and management of tuberculosis, a leading cause of death in sub-Saharan Africa [35]. Antimicrobial and antifungal effects of *C. macrostachyus* extracts have previously been reported [36, 37]. The isolated diterpenoid 12-oxo-Hardwick acid has efficacy against *Candida albicans* [38]. Ngo Bum *et al* [39] reported that decoctions of *C. macrostachyus* possess anticonvulsant effects [40]. The antimalarial efficacy of leaf and stem bark extracts of *C. macrostachyus* has been reported [41, 42]*.* Bantie *et al* [43] demonstrated a chemoprotective effect against malaria. The anthelmintic efficacy of seed extracts of *C. macrostachyus* has been reported by [44]*.* Kamanyi *et al* [45] demonstrated that extracts of stem bark of *C. macrostachyus* exhibited anti-inflammatory activity in experimental mouse models of inflammation [45]. Similar findings were also reported by Nguelefack *et al.,* (2015) [46]. Methanol leaf extract of *C. macrostachyus* showed antioxidant activity with an IC_50_ value of 0.11 mg/ml. The documented antioxidant activities of *C. macrostachyus* leaf extracts were probably due to flavonoids and phenols that have been isolated from fruits, leaves, and roots [44, 47, 48]. Flavonoids and phenolic compounds found in plants have antioxidant properties [49]. In our previous study we demonstrated that the hexane, CH_2_Cl_2_, ethyl acetate and methanol soluble fractions of a 1:1 *v/v/* CH_2_Cl_2_/MeOH crude extracts of the leaves and stem bark of *C. macrostachyus* exhibited potent anti-HIV activities against HIV-1 with IC_50_ values ranging from 0.02–8.1 μg/mL and cytotoxicity effects against MT-4 cells ranging from IC_50_ = 0.58–174 μg/mL [50]. The aim of this study was to isolate bioactive pure compounds from the hexane soluble extract of the leaves of *C. macrostachyus,* which was more potent against HIV-1 at IC_50_ = 0.02 μg/mL and analyze the cytotoxicity and anti-HIV activities of isolated pure compounds.

## Materials and methods

### General

Extraction and column chromatography (CC) was performed at the Pharmacology and Pharmacognosy department, United States International University, Kenya. Commercial silica gel (100–200, 200–300, and 300–400 mesh; Qingdao, China) was used for CC. Sephadex LH-20 (Amersham Biosciences) was also used for CC. All solvents used for CC were of analytical grade (Shanghai Chemical Reagents Co., Ltd.). CC was performed on polyamide columns (5 × 60 cm, 200 g) (Germany GmbH) over silica gel (Kieselgel 60 GF_254_, 15 μm, Merck, Germany). While Thin Layer Chromatography (TLC) was carried out on Kieselgel 60 F_254_ (Merck). Spots on UV active silica gel were detected under UV light (245 and 336 nm) and made visible using a concentrated sulphuric-anisaldehyde spray mixture and heating at 105 °C for 2 minutes. 1D and 2D NMR spectra were recorded in CDCl_3_ on a 400 MHz Bruker AVANCE NMR instrument at room temperature. Chemical shifts (δ) are expressed in ppm and were referenced against the solvent resonances at δ_H_ 7.26 and δ_C_ 77.23 ppm for ^1^H and ^13^C NMR for CDCl_3_. Structural assignments of the new compounds were made with additional information from ^1^H-^1^H COSY, HSQC, NOESY, and HMBC experiments. Mass spectra were recorded on a GC-MS Bruker MicroToF Mass Spectrometer by direct injection using a Bruker Bioapex-FTMS with electrospray ionization. The above analysis was performed at the Jodrell Laboratory, Royal Botanic Gardens Kew (UK).

### Plant material

The leaves of *C. macrostachyus* were collected from a USIU botanical garden in June 2020. The collection of the medicinal plant was performed after obtaining the required ethical approval from the Kenyatta National Hospital-University of Nairobi Ethics and Research Committee (KNH-UON ERC), approval number KNH/ERC/A/154. Taxonomic identification was done by Ms. Lucy Wambui (botanist) and voucher specimen TEREFE E. /045 was deposited for *C. macrostachyus* at the United States International University herbarium for future reference. The leaves were thoroughly washed and dried in the shade. Then, the dried leaves were pulverized using a mortar and pestle at the Medicinal Plants Laboratory of United States International University-Africa (USIU-A). The leaves were ground to a fine powder using a hammer mill.

### Extraction and isolation

The powdered plant leaves were extracted with 1:1 v/v dichloromethane: methanol solvent using the cold maceration technique. Maceration was done for 7 days with frequent agitation in an orbital shaker, and the extract was filtered. Extraction was repeated three times, and the filtrates of all portions were pooled. Finally, the extracts were concentrated using rotavapor at 30 °C to obtain dry extracts. The extract was weighed and packed in a glass vial and stored in a desiccator over silica gel until use.

**Fig. 1:**
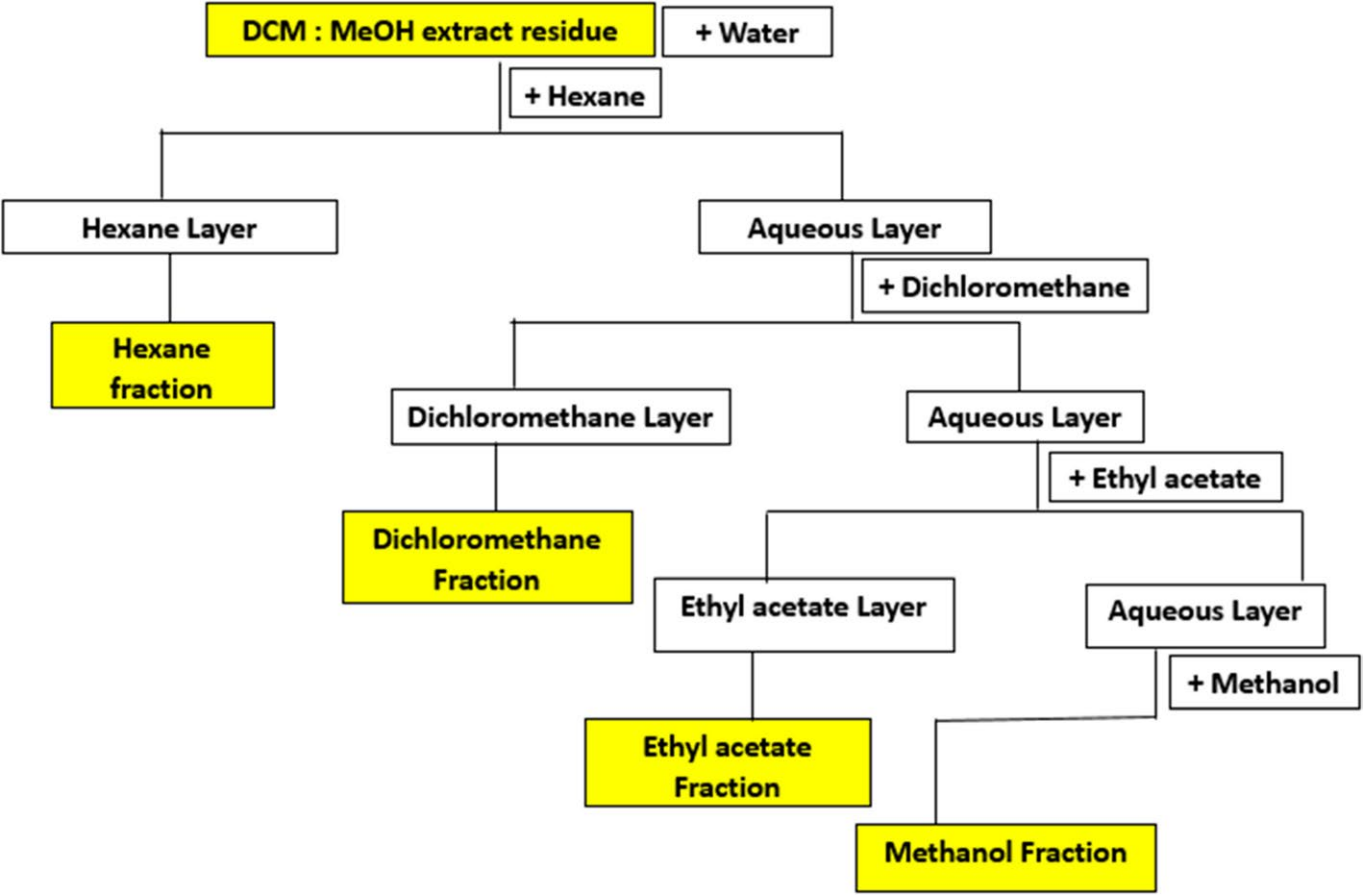
Flow chart of fractionation and isolation for activity guided isolation

The dried crude mass 1:1 v/v methanol:CH_2_Cl_2_ extract of the leaves of *C. macrostachyus* was dissolved in distilled water (200 mL) and successively partitioned using different solvents of increasing polarity (*n*-hexane, dichloromethane, ethyl acetate, and methanol) in separatory funnels (Fig. 1). The different solvent fractions were concentrated under reduced pressure using a rotary evaporator, and the resulting product was dried in an oven at 30 °C. The dried fractions were then transferred into separate vials and stored in a desiccator for further use. Bioassay-guided fractionation was performed on the partitions to determine the highest antiretroviral activity. Then, guided by their antiretroviral activity, the hexane fraction that showed the highest antiviral activity was partitioned using column chromatography, using 60–120 mesh silica gel, and eluted successively with varying concentrations of ethyl acetate and *n*-hexane (E:H). Fractions that were similar in TLC were pooled together. Each fraction was then evaluated for antiretroviral activity, and the fraction with the highest activity was further subjected to open column chromatography on 200–400 mesh silica gel. The obtain pure fractions, where necessary the fraction were subjected to column chromatography on Sephadex LH-20 as a stationary phase and methanol as the mobile phase. The eluents were monitored by thin-layer chromatography. The purity of the compounds were determined using thin-layer chromatography (TLC) on precoated aluminum-backed plates (silica gel 60 F_254,_ Merck), and compounds were visualized using UV radiation at 254 nm, followed by an anisaldehyde spray reagent (1% *p*-anisaldehyde:2% H_2_SO_4_: 97% cold MeOH) and heating. Final purifications were carried out in selected solvent systems using preparative thin-layer chromatography (Merck 818,133) and gravity column chromatography (Merck Art. 9385), which used a 2 cm diameter column packed with silica gel.

### Anti-HIV activities and cytotoxicity effects

The effects of the test compounds in preventing cytopathic effects that occur because of HIV-1 replication were evaluated by MTT colorimetric assay [51]. MT-4 cells suspended at 1 X 10^8^ cells/ml were infected with 640 μL of HIV-1_IIIB_ virus at 1.26 X10^8^ TCID50/ml. After infection, 200 μl HIV-infected MT-4 cells (1 X10^5^ cells/well) in growth media were added to each well. The plates were preincubated for 24 h at 37 °C to allow stabilization. Then, 50 μL of the test compounds (at a concentration of 4 mg/ml) were added to the first column of the well. With a multichannel pipette, 50 μL was transferred (in triplicate) from the wells labeled 1 to wells labeled 2. Such transfers were continued (serial dilution), moving from left to right, changing tips prior to mixing contents of the next column of wells. Finally, 50 μL was discarded from the wells in column 12. Different concentrations (800 to 8.192 × 105 μg/mL) of test compounds were prepared through serial dilution. Each dilution was tested in triplicates. The microtiter plates were incubated at 37 °C in a 5% CO_2_ incubator for 5 days. Two negative controls, infected untreated cells and uninfected untreated (mock) cells, and four positive controls (zidovudine, tenofovir, abacavir, and nevirapine) were also included. After 5 days of incubation, cell viability was determined by the MTT assay described [51]. All compounds were assayed in triplicate.

A dose-response curve was plotted to calculate the concentrations that reduced viral replication by 50% (IC_50_) [52–54]. The selectivity index (SI) of the test compounds was calculated as the ratio of 50% cytotoxic concentration (CC 50) to 50% effective concentration (EC50) [51].

A cytotoxicity test was conducted to evaluate the safety of the plant extracts by measuring cell death caused by the plant extracts. The assay was conducted using an MTT colorimetric assay [55, 56]. The MTT assay was based on the reduction of the yellow-colored tetrazolium salt MTT 3-(4,5-dimethylthiazol-2-yl)-2,5-diphenyltetrazolium bromide) by NAD(P)H-dependent cellular oxidoreductase enzymes [57] to an insoluble dark-blue colored formazan that can be measured spectrophotometrically [55]. Formazan production indicates the number of viable cells; therefore, an increase or decrease in cellular viability results in a change in the amount of formazan formed, which indicates the degree of cellular cytotoxicity (CC_50_) caused by the plant extract. MTT (3-(4,5-dimethylthiazol-2-yl)-2,5-diphenyl tetrazolium bromide) was dissolved in PBS to obtain a final concentration of 5 mg/ml and filtered to sterilize and remove insoluble residue [58, 59]. The assay was carried out in 96-well, flat-bottomed microtiter plates. To each well, 200 μl of MT-4 cells (1 × 10^5^ cells) in growth media was added. The plates were preincubated for 24 h at 37 °C to allow stabilization. Then, 50 μL of the test compounds (at a concentration of 4 mg/ml) were added to the first column of the well. With a multichannel pipette, 50 μL was transferred (in triplicate) from the wells labeled 1 to wells labeled 2, and such transfers were continued (serial dilution), moving from left to right, changing tips prior to mixing contents of the next column of wells. Finally, 50 μL was discarded from the wells in column 12. Different concentrations (800–8.192 × 10^5^ μg/mL) of test compounds were prepared through serial dilution. Each dilution was tested in triplicates. The negative control (NC) wells contained 50 μl of MT-4 cells in 0.5% DMSO [52]. Positive control (zidovudine, tenofovir, abacavir and nevirapine) drugs were also added in triplicate. A 96-well microtiter plate containing the test compounds and positive and negative controls was incubated at 37 °C in a humidified atmosphere of 5% CO_2_ for 5 days. After incubation, 20 μl of MTT reagent (5 mg/ml MTT in phosphate-buffered saline) was added to each test well and control well. The plate was further incubated at 37 °C in a CO_2_ incubator for 4 hours. After 4 hours of incubation, 100 μl of DMSO was added to dissolve the dark-blue formazan crystals from surviving cells [60]. After the formazan crystals were dissolved completely, the resulting optical density (OD) readings were measured relative to the controls on an ELISA plate reader at 570 nm with a reference wavelength of 620 nm [58]. For each extract and pure compound tested, 3 triplicate determinations were performed. First, the percentage viability was calculated [51] then a dose-response curve was plotted to enable the calculation of the concentrations that reduced the number of viable cells by 50% (CC50). The concentration that determined cell viability above 80% (CC20) was chosen as the maximum non-toxic concentration (MNTC).

## Results

### Hexane soluble extract of 1:1 *v/v/* CH_2_Cl_2_/MeOH crude extract of the leaves of *C. macrostachyus*

**Fig. 2:**
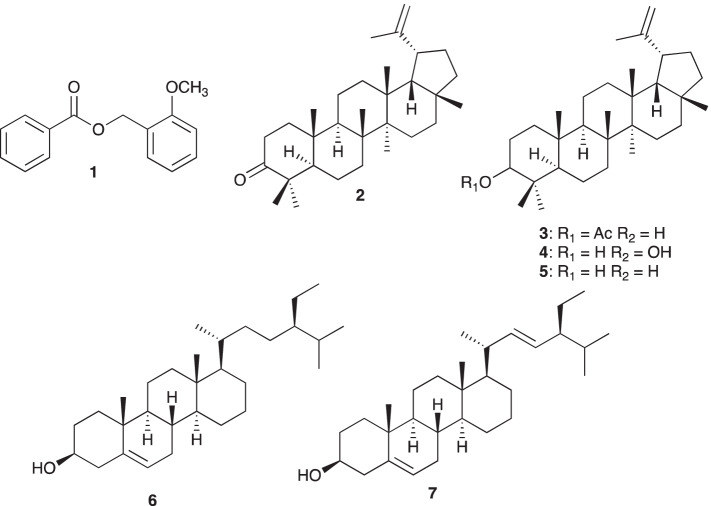
Structures of isolated compounds from *C. macrostachyus*

Repeated column chromatography and preparative thin-layer chromatography of hexane soluble fraction of the leaves of *C. macrostachyus* afforded 2-methoxy benzyl benzoate (1), lupenone (2), lupeol acetate (3), betulin (4), lupeol (5), sitosterol (6) and stigmasterol (7) (Fig. 2). The NMR spectrum of these compounds is available in the supplementary information.

### Anti-HIV activities and cytotoxicity effects of the isolated compounds

**Fig. 3:**
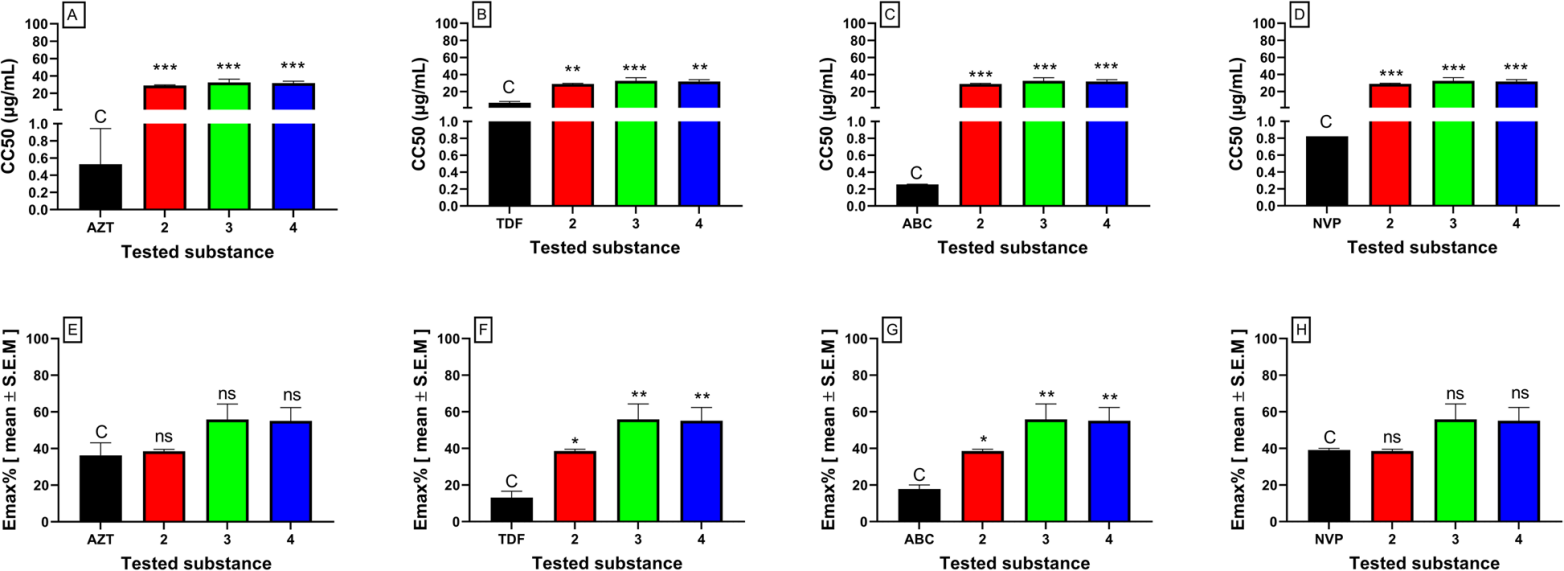
Cytotoxicity of pure compounds isolated from *C. macrostachyus.* The results are expressed as the mean of three independent experiments ± S.E.M. AZT, Zidovudine; TDF, Tenofovir; ABC, Abacavir; NVP, Nevirapine; Lupenone (2); Lupeol acetate (3); Betulin (4); CC50, 50% cytotoxic concentration; EmaxC, Maximum cytotoxic effect; C; control, ns, not significant, *Denotes *p* value < 0.05; **Denotes *p* value < 0.01, ***Denotes *p* value < 0.001

**Fig. 4:**
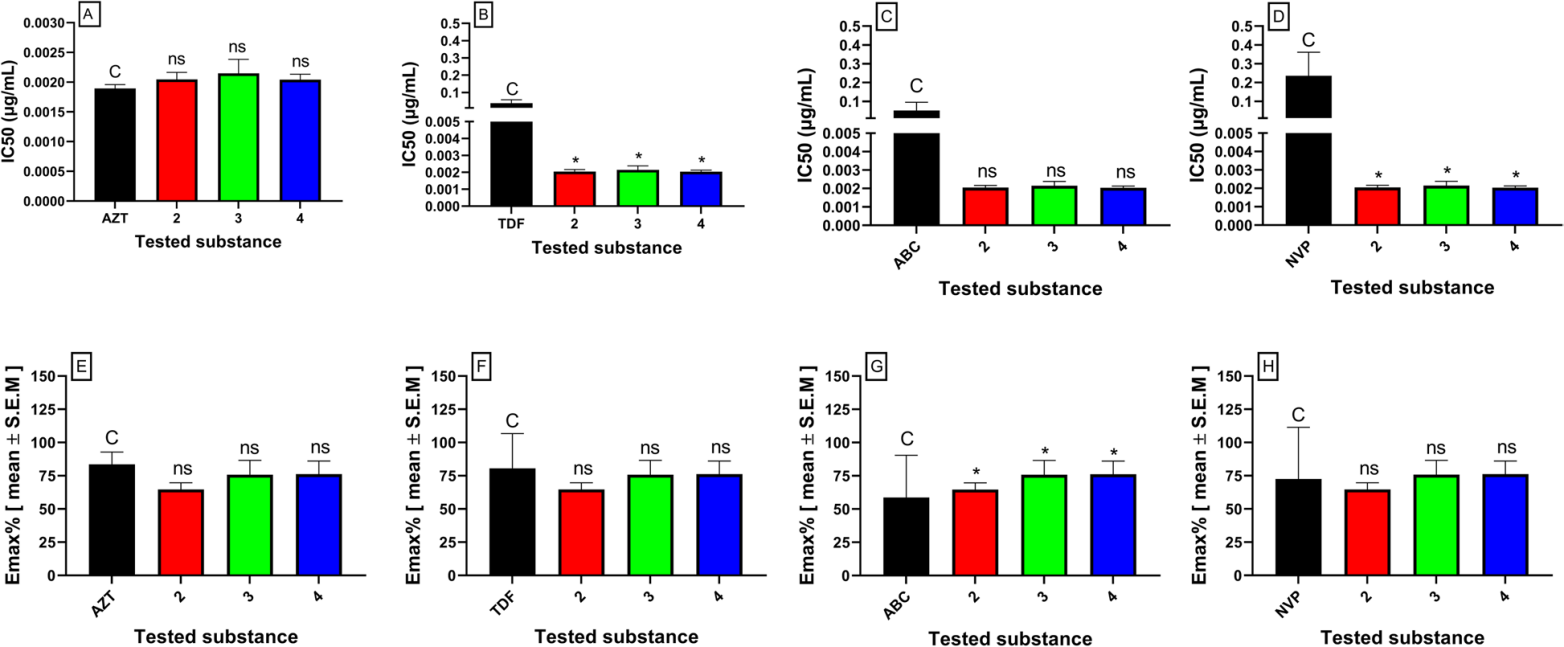
Anti-HIV activity of pure compounds isolated from *C. macrostachyus****.*** The results are expressed as the mean of three independent experiments ± S.E.M. AZT, Zidovudine; TDF, Tenofovir; ABC, Abacavir; NVP, Nevirapine; Lupenone (2); Lupeol acetate (3); Betulin (4); CC_50_, 50% cytotoxic concentration; Emax_C_, Maximum cytotoxic effect; C; control, ns, not significant, *Denotes *p* value < 0.05; **Denotes *p* value < 0.01, ***Denotes *p* value < 0.001

**Fig. 5:**
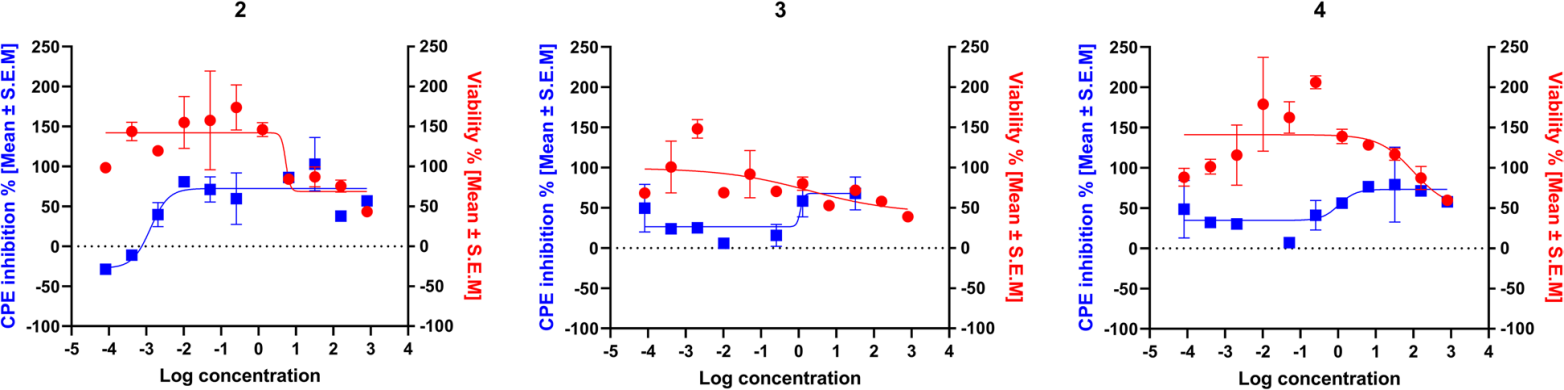
Concentration-response curve analysis for the anti-HIV activity of pure compounds isolated from *C. macrostachyus.* Results presented in the curves are means ± S.E.M of three independent experiments. Cell viability % (red line) and the inhibition % of the virus-induced cytopathic effect (blue line) associated with control drugs and the tested extracts at the concentration level (800–8.192 × 10^5^ μg/mL); Lupenone (2); Lupeol acetate (3); Betulin (4)

The cytotoxic and antiviral activity findings for the pure compounds isolated from *C. macrostachyus* and the control drugs are summarized in Table 1. Among the three isolated compounds from *C. macrostachyus,* lupenone (2) displayed the highest CC_50_ value of 32.46 ± 0.7 μg/mL (Table 1). Furthermore, a comparison between the pure isolated compounds and the control drugs showed that compounds 2, 3 and 4 had significantly (*p* < 0.001) higher CC_50_ values than AZT, ABC, and NVP, which indicates their safety and that high concentration levels are required to exert cytotoxic effects (Fig. 3). In addition, the maximum cytotoxic effect (Emax_c_) of the compounds was not significantly different from the cytotoxic effect of AZT and NVP. Betulin (4) observed the highest anti-HIV activity, which inhibited virus-induced CPE by 76% with an IC_50_ value of 0.002 ± 0.04 μg/mL, which is much lower than the maximum nontoxic concentration (MNTC), which also indicates the safety and efficacy of the compound. Furthermore, all three compounds displayed anti-HIV activity at significantly (*p* < 0.05) lower IC_50_ values than TDF and NVP (Fig. 3), indicating their higher potency. In addition, the three compounds displayed significantly (*p* < 0.05) higher inhibition of viral-induced CPE (Emax_AV_) than ABC. The antiviral activity of the tested compounds (Emax_AV_) showed that both the control drugs (except ABC) and the tested compounds had approximately similar antiviral efficacy, as they showed non-significantly different Emax_AV_ values (Fig. 4). Furthermore, the results showed that the tested compounds had a higher selectivity index, indicating their efficacy at lower cytotoxic effects. As depicted in Fig. 5, the pure compounds (2, 3, and 4) displayed concentration-dependent inhibition of virus-induced CPE, as the % CPE inhibition increased with increasing concentrations of the pure compounds. Our finding on the anti-HIV activity of these pure compounds is in agreement with previous reports. A report by Chaniad *et al* [61] explained the efficacy of betulin (4) as a potent anti-HIV compound with an IC_50_ value of 17.7 ± 0.6 μM. Similarly, Esposito *et al* [62] reported that lupeol acetate and lupeol inhibited HIV-1 RT-associated RNase H function with IC_50_ values of 63 and 11.6 μM, respectively.

**Table 1:** Cytotoxicity and anti-HIV activities of pure compounds isolated from *C. macrostachyus*

Tested compounds	Cytotoxicity			Antiviral activity		SI
	MNTC (μg/mL)	CC_**50**_ (μg/mL)	Emax_**C**_ (%)	IC_**50**_ (μg/mL)	Emax_**AV**_ (%)	
**FDA Approved antiretroviral Drugs**						
**AZT**	0.38 ± 0.19	0.53 ± 0.29	36.28 ± 0.83	0.002 ± 0.00	83.5 ± 0.57	279.4
**TDF**	4.92 ± 0.71	6.73 ± 0.24	13.17 ± 0.43	0.04 ± 0.01	80.55 ± 0.46	176.5
**ABC**	0.18 ± 0.03	0.26 ± 0.00	17.83 ± 0.57	0.05 ± 0.031	58.67 ± 0.43	5.0
**NVP**	0.57 ± 0.0	0.82 ± 0.0	39.13 ± 0.65	0.24 ± 0.09	72.53 ± 0.47	3.5
**Pure compounds isolated from** ***C. macrostachyus***						
**1**	0.002 ± 0.00	0.001 ± 0.00	39.1 ± 2.22	0.25 ± 0.02	53.22 ± 3.345	0.0073
**2**	14.17 ± 0.94	28.83 ± 0.54	38.53 ± 0.69	0.002 ± 0.001	64.74 ± 0.52	14,084.0
**3**	16.01 ± 0.64	32.46 ± 0.7	55.91 ± 0.93	0.002 ± 0.00	75.8 ± 0.59	15,097.7
**4**	16.54 ± 0.35	31.74 ± 0.55	55.13 ± 0.15	0.002 ± 0.04	76.17 ± 0.02	15,551.2
**5**	75.75 ± 0.74	141.8 ± 0.7	70.28 ± 0.75	0.047 ± 0.02	77.01 ± 0.38	3048.2
**6**	4.41 ± 0.04	5.83 ± 0.20	42.74 ± 0.09	5.58 ± 0.231	84.65 ± 0.54	1.0
**7**	4.70 ± 3.43	12.38 ± 10.88	43.71 ± 2.02	0.14 ± 0.04	76.77 ± 23.24	86.5

Results are shown as mean ± S.E.M (*n* = 3)

*AZT* Zidovudine, *TDF* Tenofovir, *ABC* Abacavir, *NVP* Nevirapine; 2-methoxy benzyl benzoate (1); lupenone (2); lupeol acetate (3); betulin (4); lupeol (5); sitosterol (6); stigmasterol (7); *MNTC* Maximum nontoxic concentration, *CC*_*50*_ 50% cytotoxic concentration, *Emax*_*C*_ Maximum cytotoxic effect %, *IC*_*50*_ 50% antiviral effect concentrations, *Emax*_*AV*_ Maximum antiviral effect %, *SI* Selective index

## Conclusions

We conclude that the hexane soluble extract of 1:1 *v/v/* CH_2_Cl_2_/MeOH crude extract of the leaves of *C. macrostachyus,* is potent against HIV-1 at IC_50_ = 0.02 μg/mL, and that 2-methoxy benzyl benzoate (1), lupenone (2), lupeol acetate (3), betulin (4), lupeol (5), sitosterol (6) and stigmasterol (7) are its major constituents. We demonstrated that lupenone (2), lupeol acetate (3) and betulin (4) exhibited anti-HIV-1 inhibition at 4.7, 4.3 and 4.5 nM respectively. These results are in agreement with previously reported ant-HIV activities of the known compounds. Chaniad *et al* [61] described the efficacy of betulin (4) against HIV with an IC_50_ value of 17.7 ± 0.6 μM, whereas Esposito *et al* [62] reported that lupeol acetate and lupeol inhibited HIV-1 RT-associated RNase H function with IC_50_ values of 63 and 11.6 μM, respectively. The current results and the described past anti-HIV effects of compounds found in *C. macrostachys*, ascertains the importance of the plant, as was previously reported by Maroyi (2017) [18].

## Supplementary Information

**Additional file 1.**

## Data Availability

All data generated or analysed during this study are included in this published article and its supplementary information files.

## Abbreviations

ABC: Abacavir
AZT: Zidovudine
CC_50_: 50% cytotoxic concentration
CDD: Methylene chloride soluble fractions of 1:1 CH_2_Cl_2_: MeOH extract
CDE: Ethyl acetate soluble fractions of 1:1 CH_2_Cl_2_: MeOH extract
CDH: Hexane soluble fractions of 1:1 CH_2_Cl_2_: MeOH extract
CDM: Methanol soluble fractions of 1:1 CH_2_Cl_2_: MeOH extract
Emax_AV_: Maximum antiviral effect %
Emax_C_: Maximum cytotoxic effect %
IC_50_: 50% antiviral effect concentration
MNTC: Maximum non-toxic concentration
NVP: Nevirapine
SI: selectivity index
