# Supplementary material for: In vitro anti-HIV and cytotoxic effects of pure compounds isolated from Croton macrostachyus Hochst. Ex Delile
Source: BMC Complement Med Ther. 2022 Jun 15;22:159. doi: 10.1186/s12906-022-03638-6 (PMC9202147; doi:10.1186/s12906-022-03638-6)
Supplement: Supplementary file 1 — Additional file 1. [file 12906_2022_3638_MOESM1_ESM.docx]

*In vitro* anti-HIV and cytotoxic effects of pure compounds isolated from *Croton macrostachyus* Hochst. Ex Delile

**Ermias Mergia Terefe ^1,2,^*, Faith A. Okalebo^2^ , Solomon Derese^3^, Moses K Langat ^4^, Eduard Mas-Claret^4^, Nada H. Aljarba^5^, Saad Alkahtani^6^, Gaber El-Saber Batiha^7^, Arabinda Ghosh^8^**, **Eman A. El-Masry^9^, and Joseph Muriuki^10^**

^1^ Department of Pharmacology and Pharmacognosy, School of Pharmacy and Health Sciences, United States International University-Africa, P.O. BOX 14634 – 00800, Nairobi, Kenya; [eterefe@usiu.ac.ke](mailto:eterefe@usiu.ac.ke)

^2^ Department of Pharmacology and Pharmacognosy, College of Health Sciences, University of Nairobi, Nairobi, Kenya; [faith.okalebo@uonbi.ac.ke;](mailto:faith.okalebo@uonbi.ac.ke) [eterefe@usiu.ac.ke](mailto:eterefe@usiu.ac.ke)

^3^ Department of Chemistry, University of Nairobi, Nairobi, Kenya; [sderese@uonbi.ac.ke](mailto:sderese@uonbi.ac.ke)

^4^ Royal Botanic Gardens, Kew, Kew Green, Richmond, Surrey, TW9 3AE, UK; [m.langat@kew.org](mailto:m.langat@kew.org); [e.masclaret@kew.org](mailto:e.masclaret@kew.org)

^5^ Department of Biology, College of Science, Princess Nourah bint Abdulrahman University, P. O. Box 84428, Riyadh 11671, Saudi Arabia. [nhaljarba@pnu.edu.sa](mailto:nhaljarba@pnu.edu.sa)

^6^ Department of Zoology, College of Science, King Saud University, P. O. Box 2455, Riyadh 11451, Saudi Arabia. [salkahtani@ksu.edu.sa](mailto:salkahtani@ksu.edu.sa)

^7^ Department of Pharmacology and Therapeutics, Faculty of Veterinary Medicine, Damanhour University, Damanhour 22511, AlBeheira, Egypt; [gaberbatiha@gmail.com](mailto:gaberbatiha@gmail.com)

^8^ Microbiology Division, Department of Botany, Gauhati University, Guwahati, Assam-781014, India; [dra.ghosh@gauhati.ac.in](mailto:dra.ghosh@gauhati.ac.in)

^9^ Microbiology and Immunology unit, Department of Pathology, College of Medicine, Jouf University, Al-Jouf,Sakaka, Saudi Arabia, [ealmasry@ju.edu.sa](mailto:ealmasry@ju.edu.sa)

^10^ Centre for Virus Research, Kenya Medical Research Institute, Nairobi, Kenya; [jmuriuki@kemri.org](mailto:jmuriuki@kemri.org)

***** Correspondence: [eterefe@usiu.ac.ke](mailto:eterefe@usiu.ac.ke); Tel.: +254746272742

**Table of content**

[**Figure 1 ^1^H NMR spectra of 2-Methoxy benzyl benzoate  (1)** 4](#_Toc103621616)

[**Figure 2 ^13^C NMR spectra of 2-Methoxy benzyl benzoate (2)** 5](#_Toc103621617)

[**Figure 3 DEPT spectrum of 2-Methoxy benzyl benzoate (1)** 6](#_Toc103621618)

[**Figure 4 HSQCDEPT spectrum of 2-Methoxy benzyl benzoate (1)** 7](#_Toc103621619)

[**Figure 5 HMBC spectrum of 2-Methoxy benzyl benzoate (1)** 8](#_Toc103621620)

[**Figure 6 COSY spectrum of 2-Methoxy benzyl benzoate  (1)** 9](#_Toc103621621)

[**Figure 7 ^1^H NMR spectrum of Lupenone (2)** 10](#_Toc103621622)

[**Figure 8 ^13^C NMR spectrum of Lupenone (2)** 11](#_Toc103621623)

[**Figure 9 DEPT spectrum of Lupenone (2)** 12](#_Toc103621624)

[**Figure 10 HSQCDEPT spectrum of Lupenone (2)** 13](#_Toc103621625)

[**Figure 11 HMBC spectrum of Lupenone (2)** 14](#_Toc103621626)

[**Figure 12 COSY spectrum of Lupenone (2)** 15](#_Toc103621627)

[**Figure 13 NOESY spectrum of Lupenone (2)** 16](#_Toc103621628)

[**Figure 14 ^1^H NMR spectrum of Lupeol acetate (3)** 17](#_Toc103621629)

[**Figure 15 ^13^C NMR spectrum of Lupeol acetate (3)** 18](#_Toc103621630)

[**Figure 16 DEPT spectrum of Lupeol acetate (3)** 19](#_Toc103621631)

[**Figure 17 HSQCDEPT spectrum of Lupeol acetate (3)** 20](#_Toc103621632)

[**Figure 18 HMBC spectrum of Lupeol acetate (3)** 21](#_Toc103621633)

[**Figure 19 COSY spectrum of Lupeol acetate (3)** 22](#_Toc103621634)

[**Figure 20 NOSEY spectrum of Lupeol acetate (3)** 23](#_Toc103621635)

[**Figure 21 ^1^H NMR spectrum of Betulin (4)** 24](#_Toc103621636)

[**Figure 22 ^13^C NMR spectrum of Betulin (4)** 25](#_Toc103621637)

[**Figure 23 DEPT spectrum of Betulin (4)** 26](#_Toc103621638)

[**Figure 24 HSQCDEPT spectrum of Betulin (4)** 27](#_Toc103621639)

[**Figure 25 HMBC spectrum of Betulin (4)** 28](#_Toc103621640)

[**Figure 26 COSY spectrum of Betulin (4)** 29](#_Toc103621641)

[**Figure 27 NOSEY spectrum of Betulin (4)** 30](#_Toc103621642)

[**Figure 28 ^1^H NMR spectrum of Lupeol (5)** 31](#_Toc103621643)

[**Figure 29 ^13^C NMR spectrum of Lupeol (5)** 32](#_Toc103621644)

[**Figure 30 DEPT spectrum of Lupeol (5)** 33](#_Toc103621645)

[**Figure 31 HSQCDEPT spectrum of Lupeol (5)** 34](#_Toc103621646)

[**Figure 32 HMBC spectrum of Lupeol (5)** 35](#_Toc103621647)

[**Figure 33 COSY spectrum of Lupeol (5)** 36](#_Toc103621648)

[**Figure 34 NOESY spectrum of Lupeol (5)** 37](#_Toc103621649)

[**Figure 35 ^1^H NMR Sitosterol (6) and stigmasterol (7)** 38](#_Toc103621650)

# **Figure 1 ^1^H NMR spectra of 2-Methoxy benzyl benzoate  (1)**

**
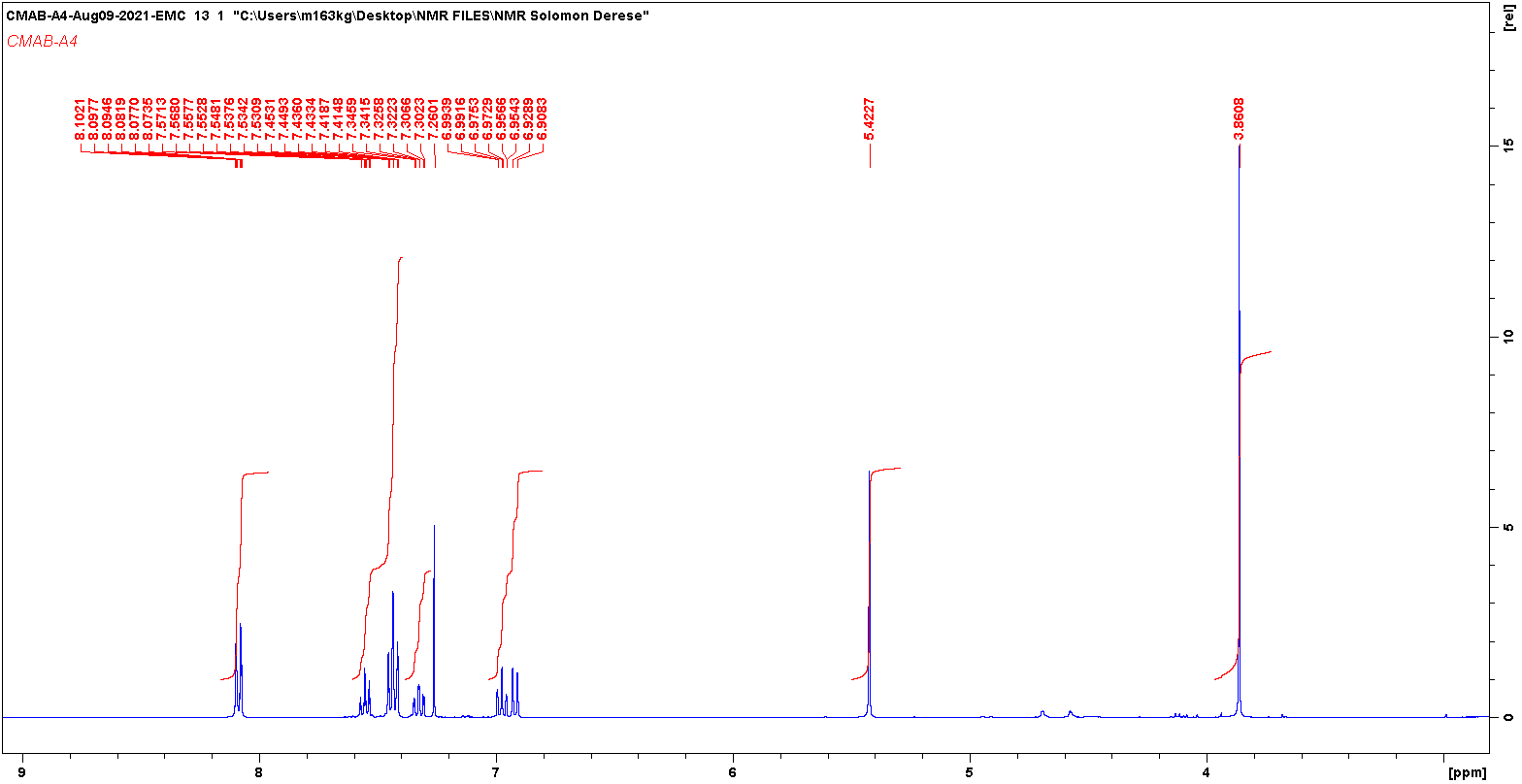
**

# **Figure 2 13C NMR spectra of 2-Methoxy benzyl benzoate (2)**

**
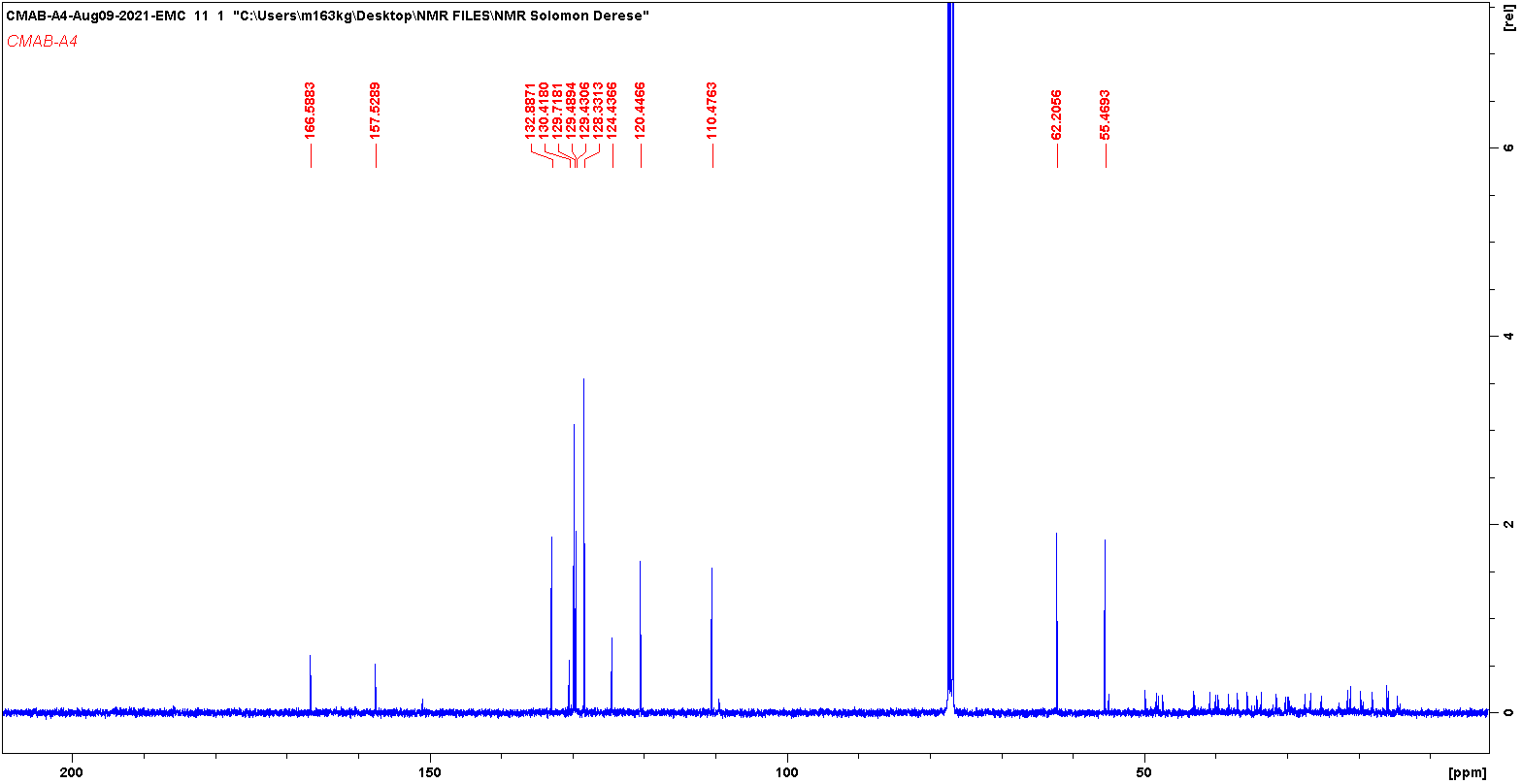
**

# **Figure 3 DEPT spectrum of 2-Methoxy benzyl benzoate (1)**

**
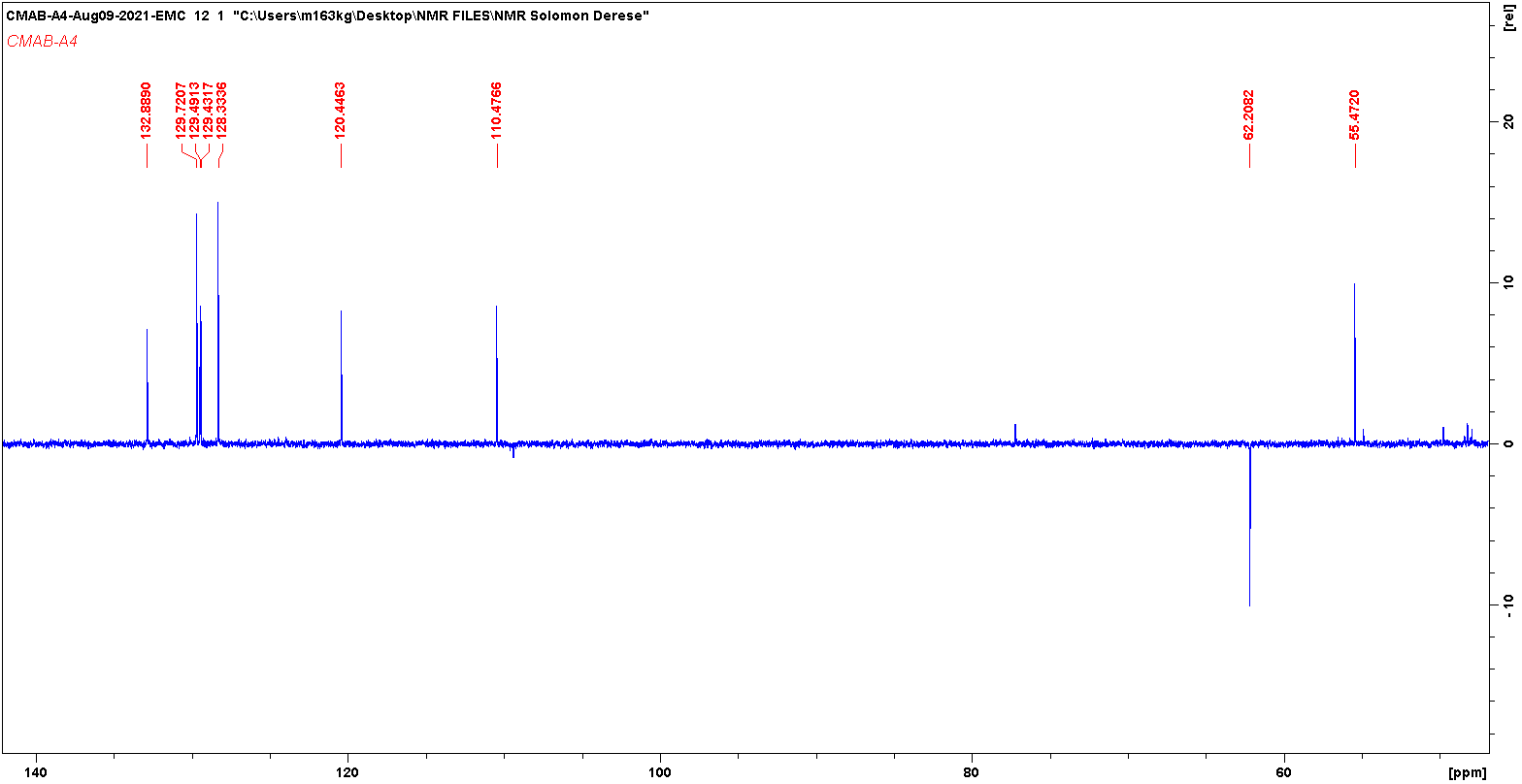
**

# **Figure 4 HSQCDEPT spectrum of 2-Methoxy benzyl benzoate (1)**

**
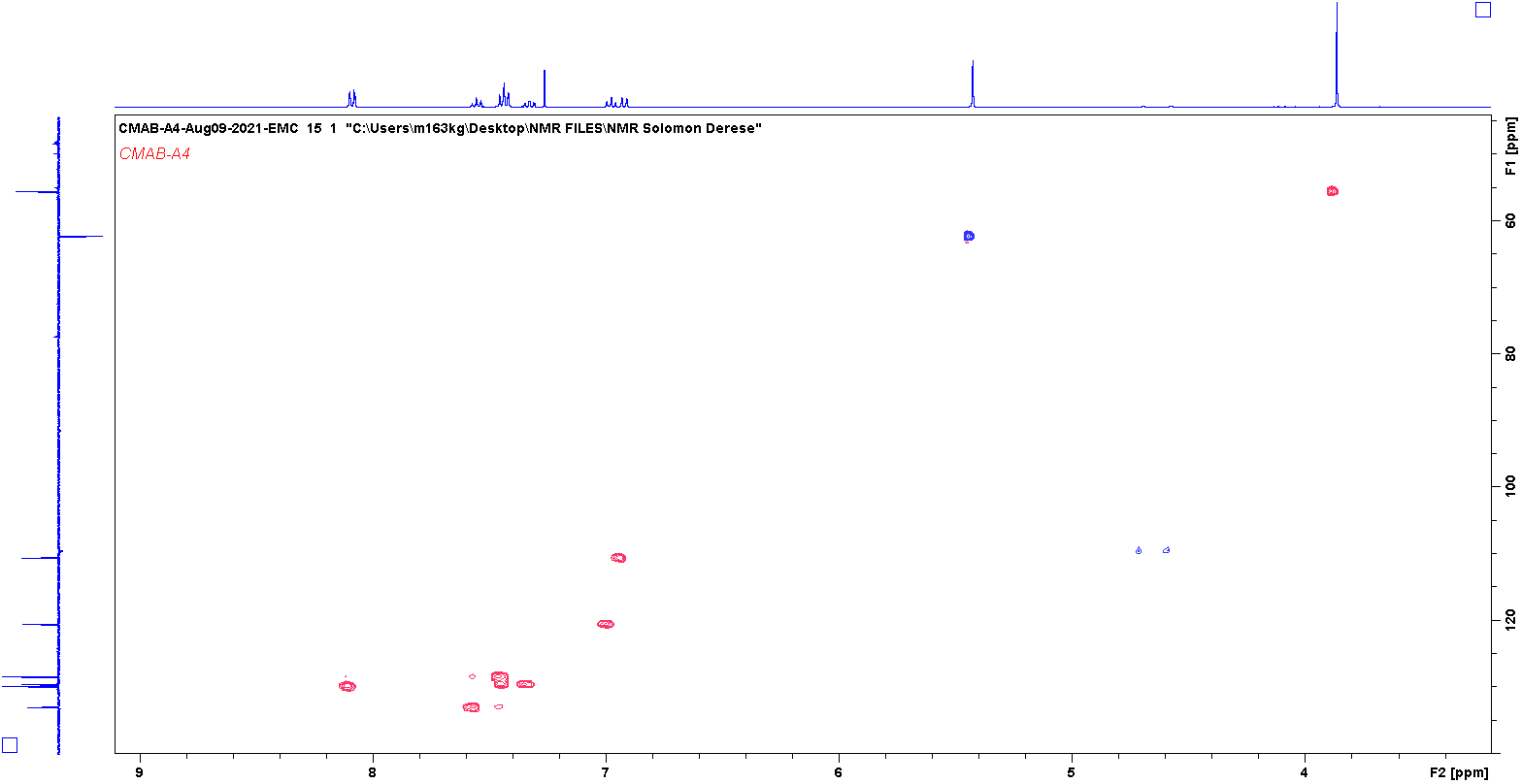
**

# **Figure 5 HMBC spectrum of 2-Methoxy benzyl benzoate (1)**

**
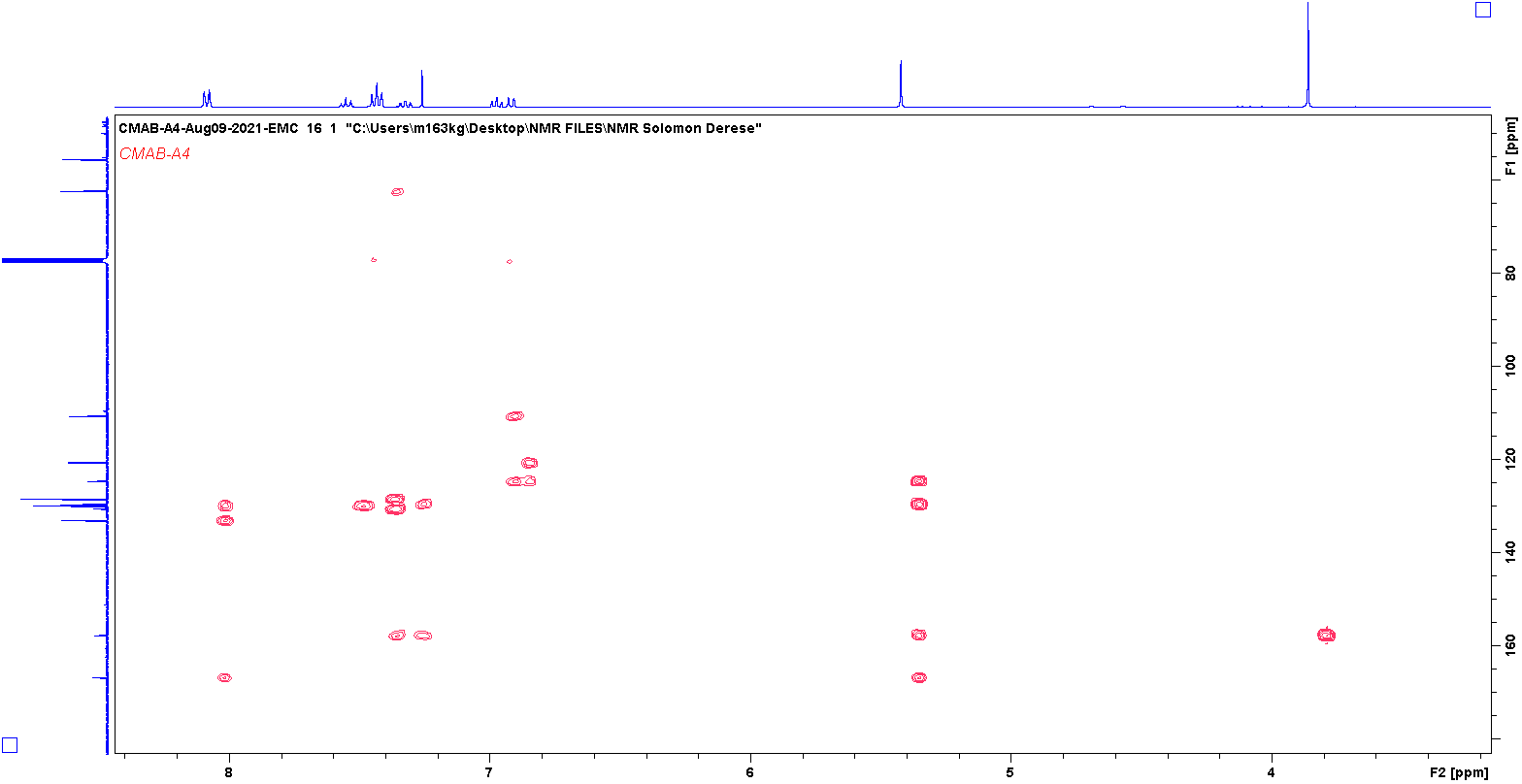
**

# **Figure 6 COSY spectrum of 2-Methoxy benzyl benzoate  (1)**

**
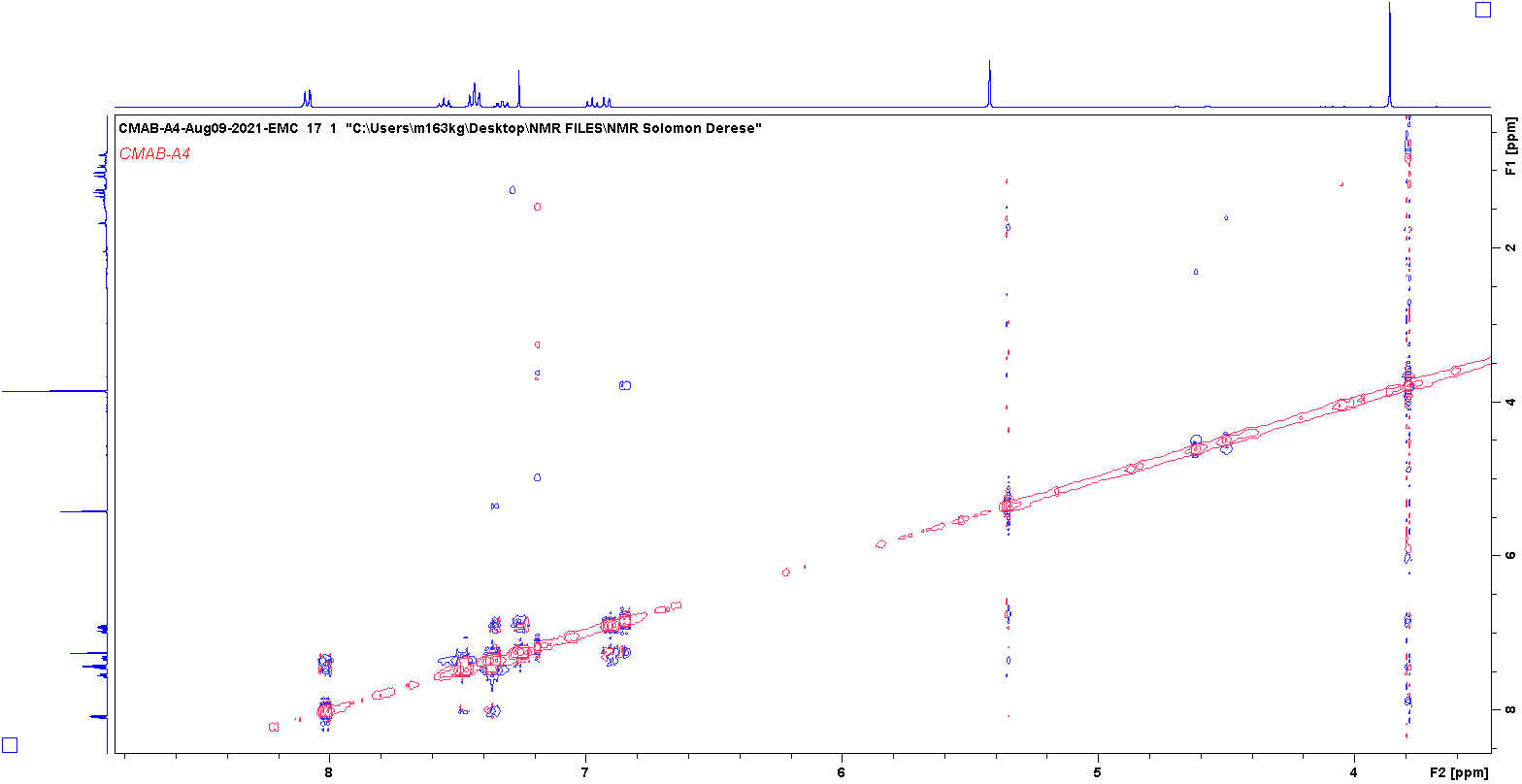
**

# **Figure 7 1H NMR spectrum of Lupenone (2)**

**
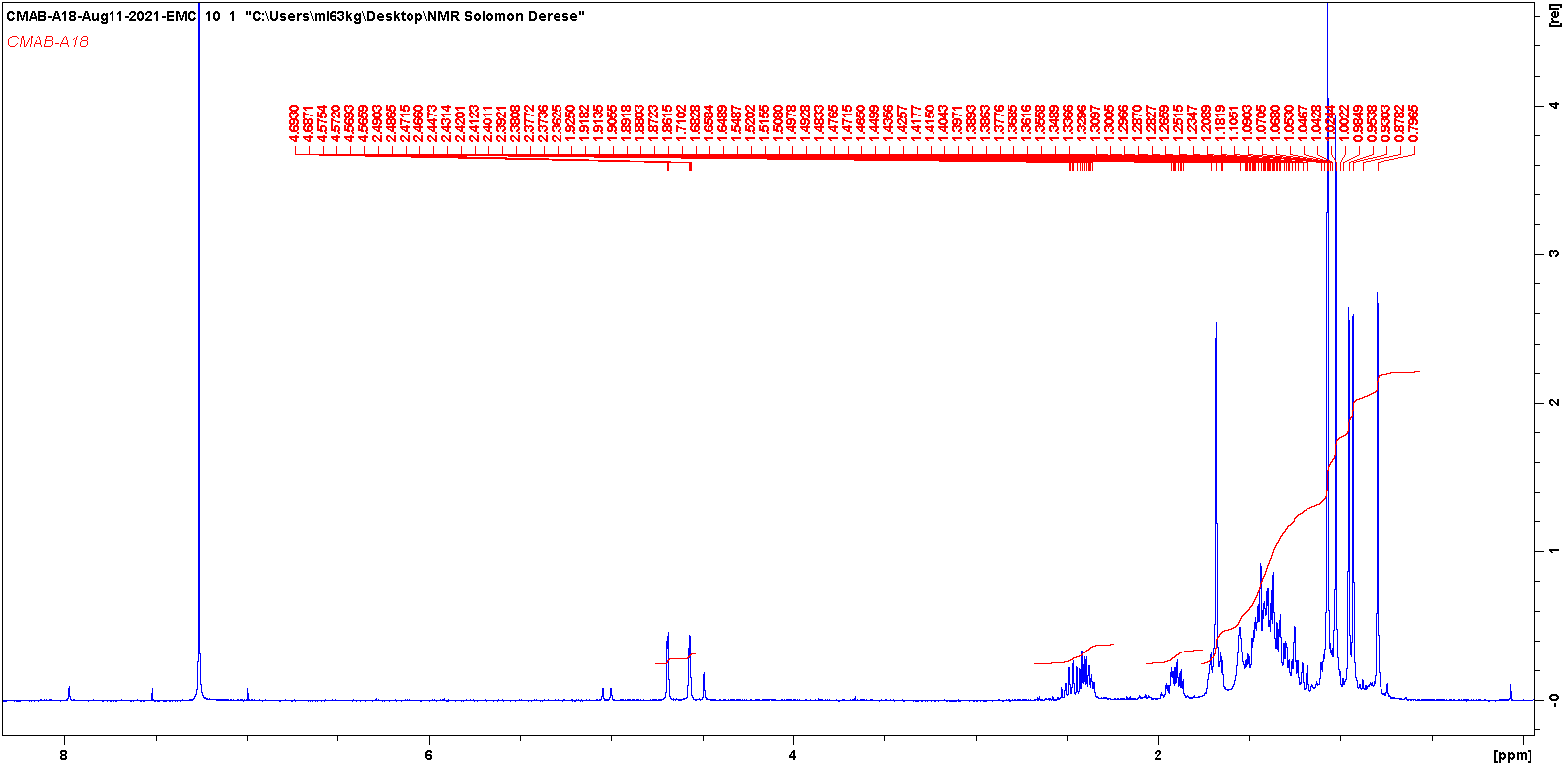
**

# **Figure 8 13C NMR spectrum of Lupenone (2)**

**
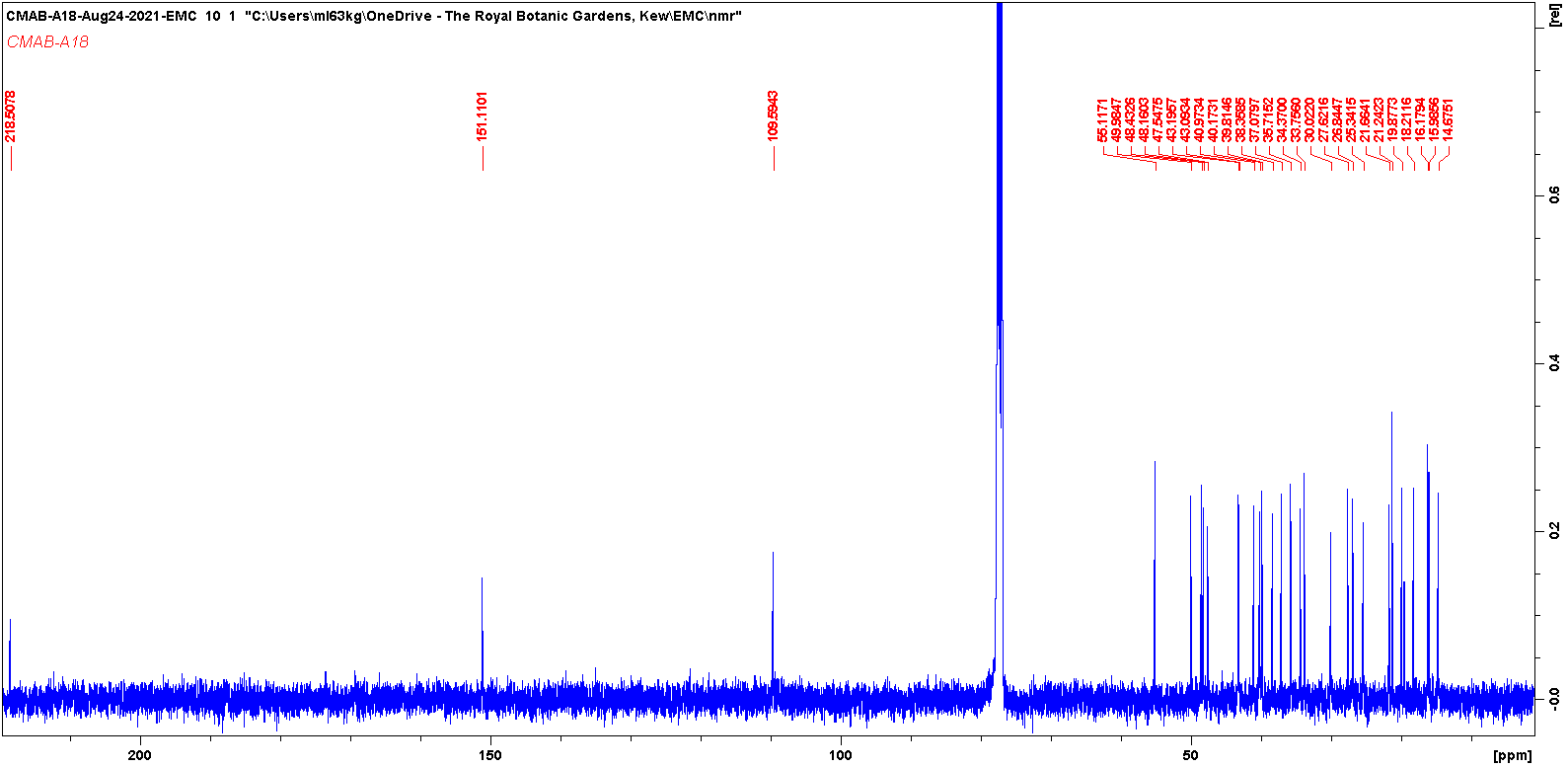
**

# **Figure 9 DEPT spectrum of Lupenone (2)**

**
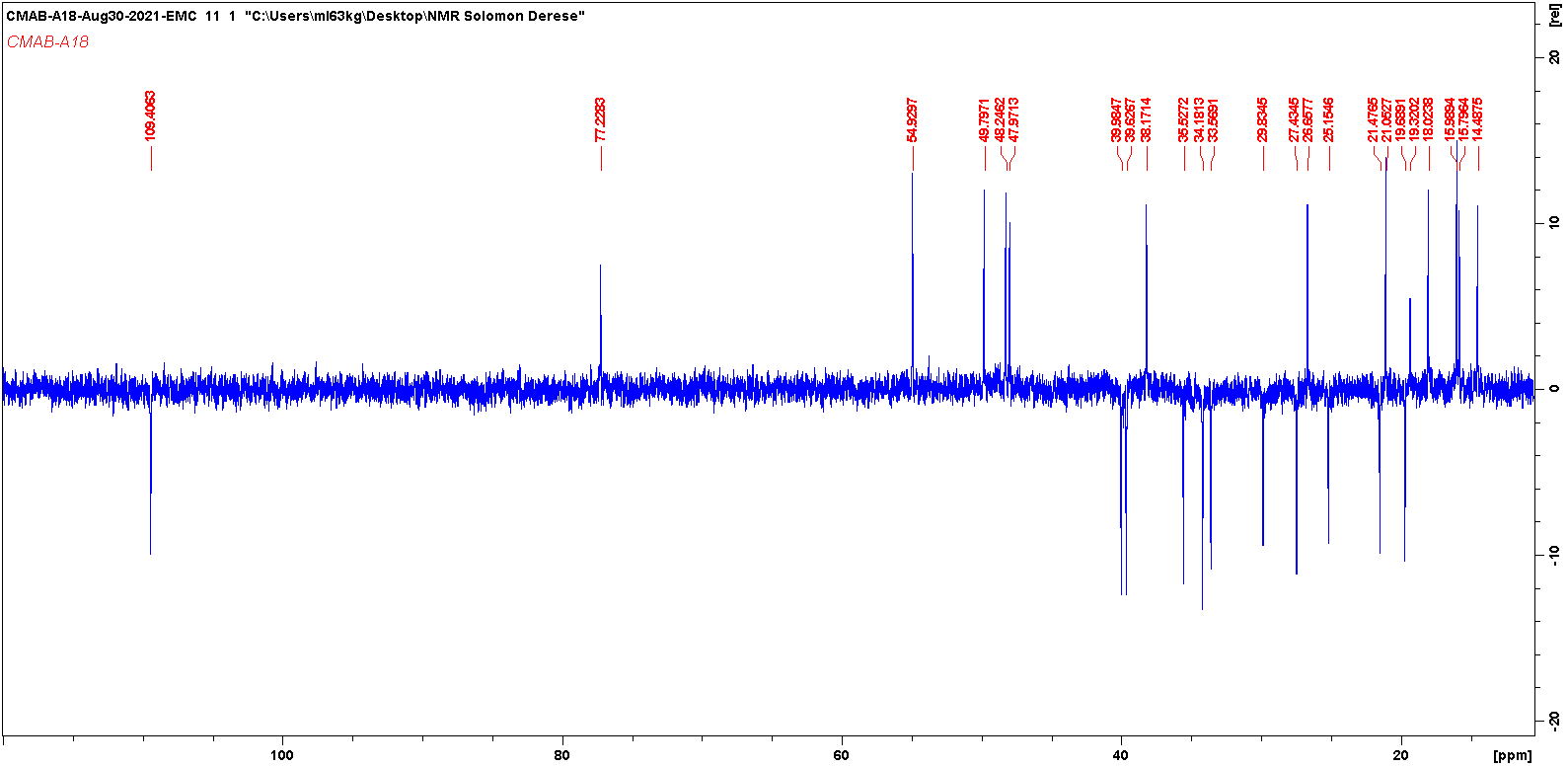
**

# **Figure 10 HSQCDEPT spectrum of Lupenone (2)**

**
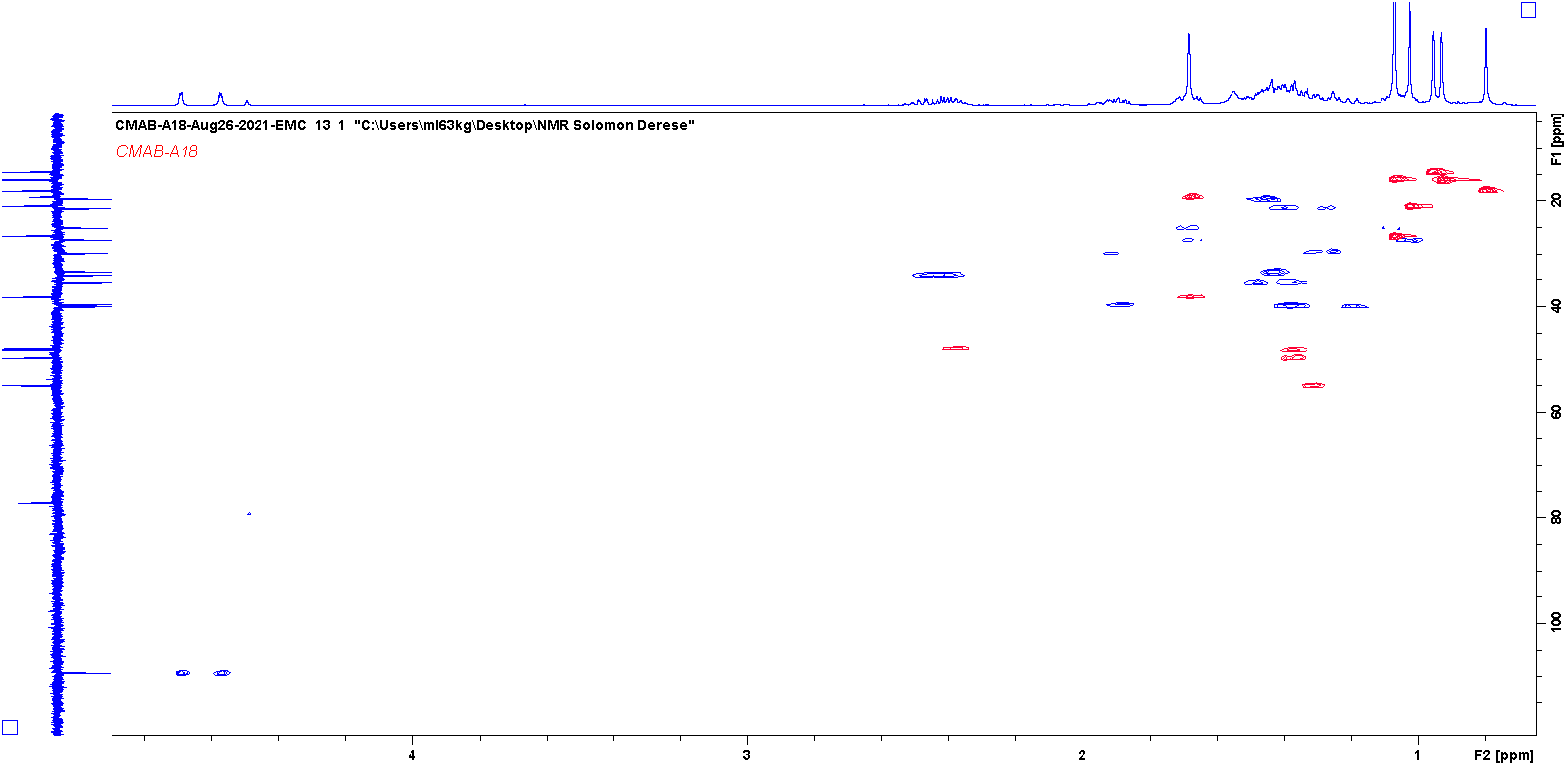
**

# **Figure 11 HMBC spectrum of Lupenone (2)**

**
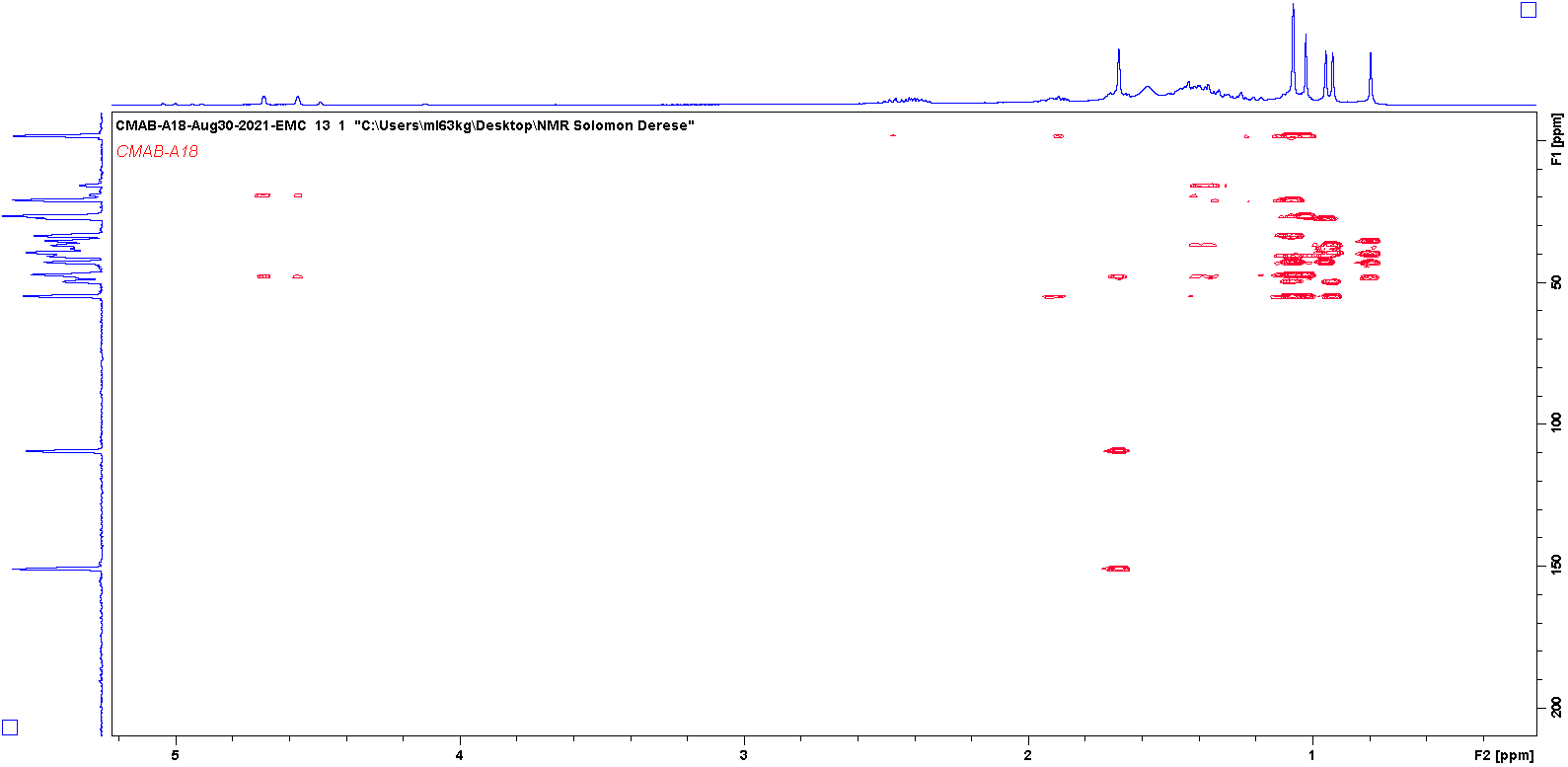
**

# **Figure 12 COSY spectrum of Lupenone (2)**

**
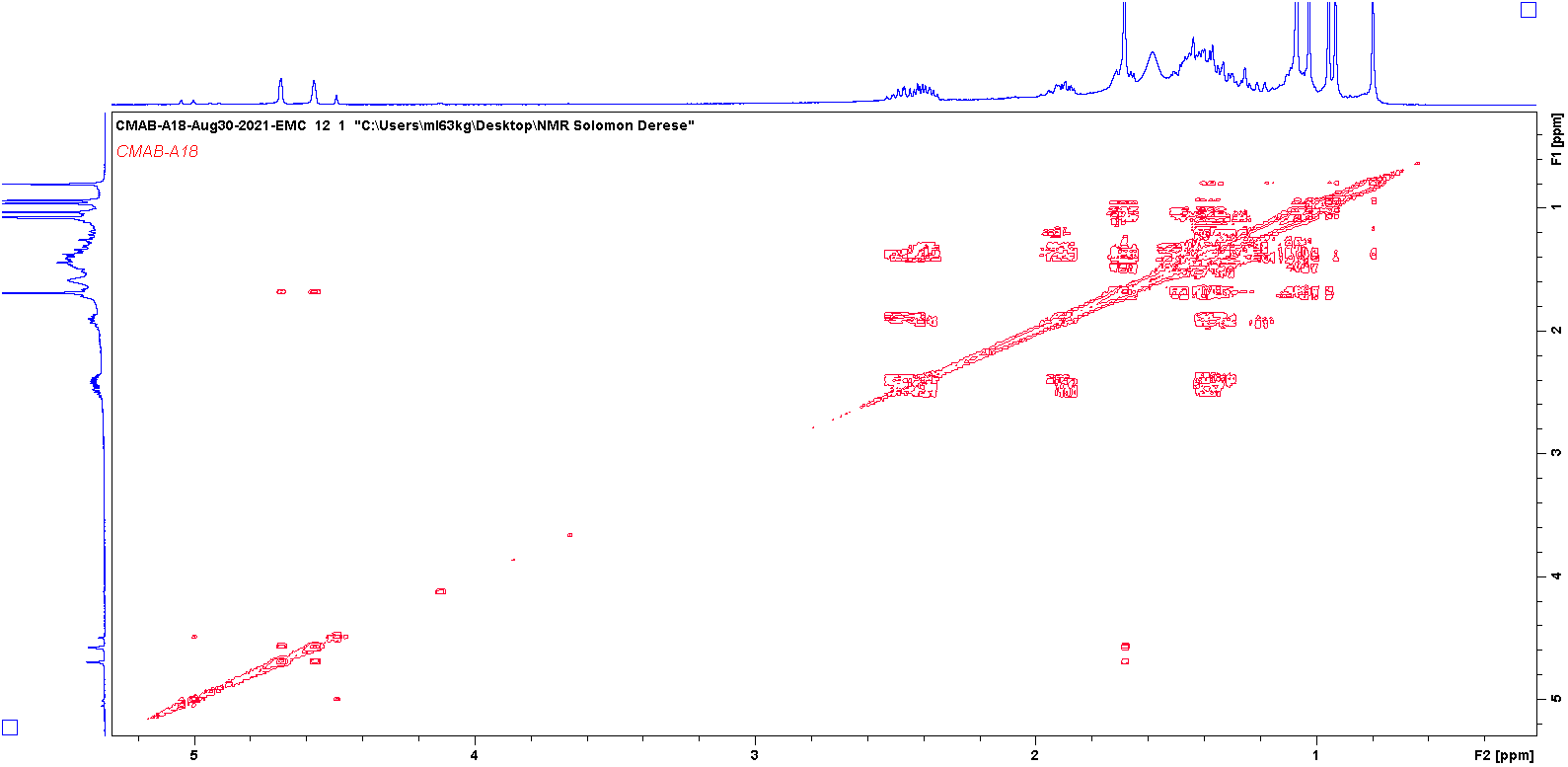
**

# **Figure 13 NOESY spectrum of Lupenone (2)**

**
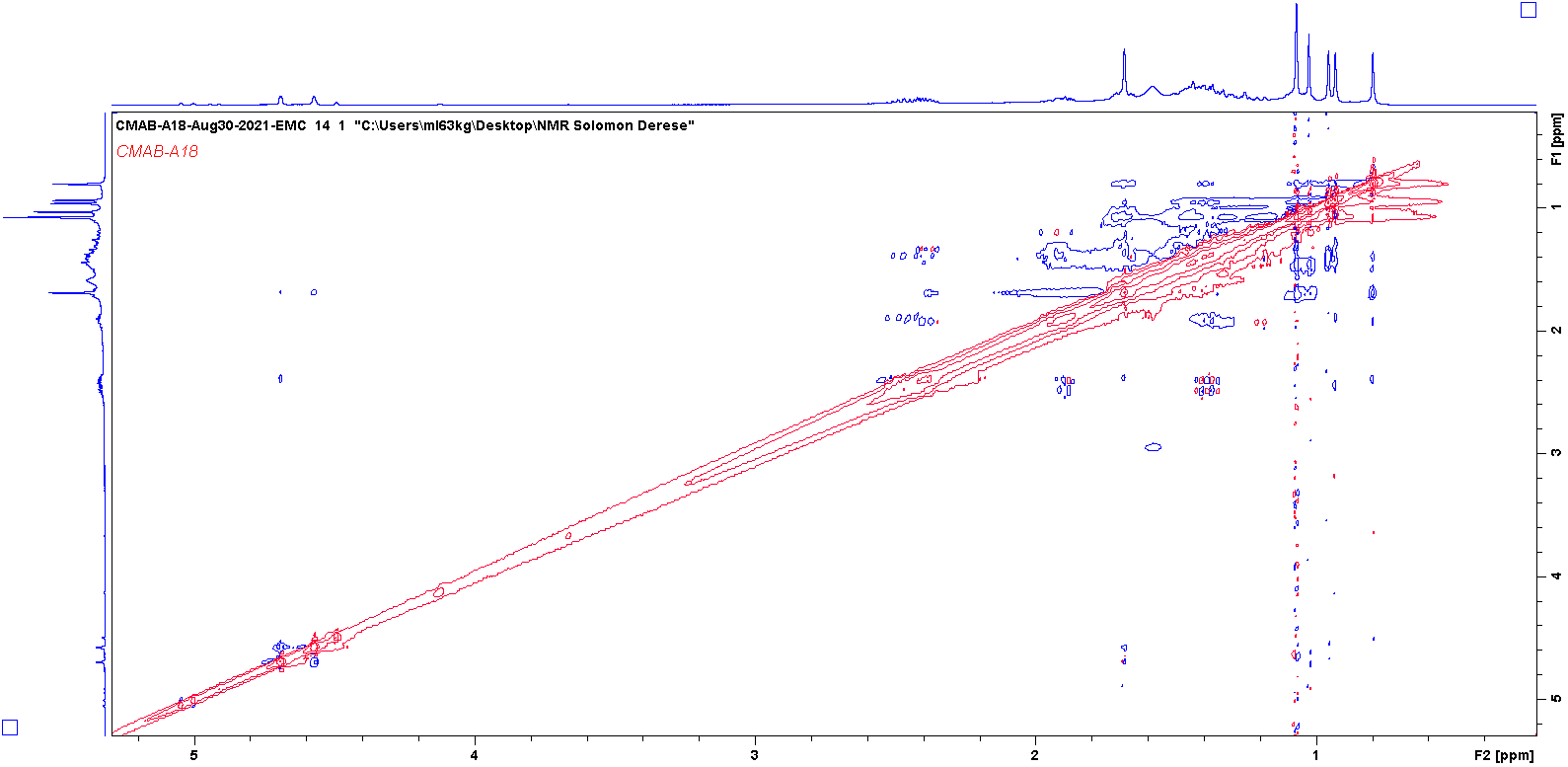
**

# **Figure 14 1H NMR spectrum of Lupeol acetate (3)**

**
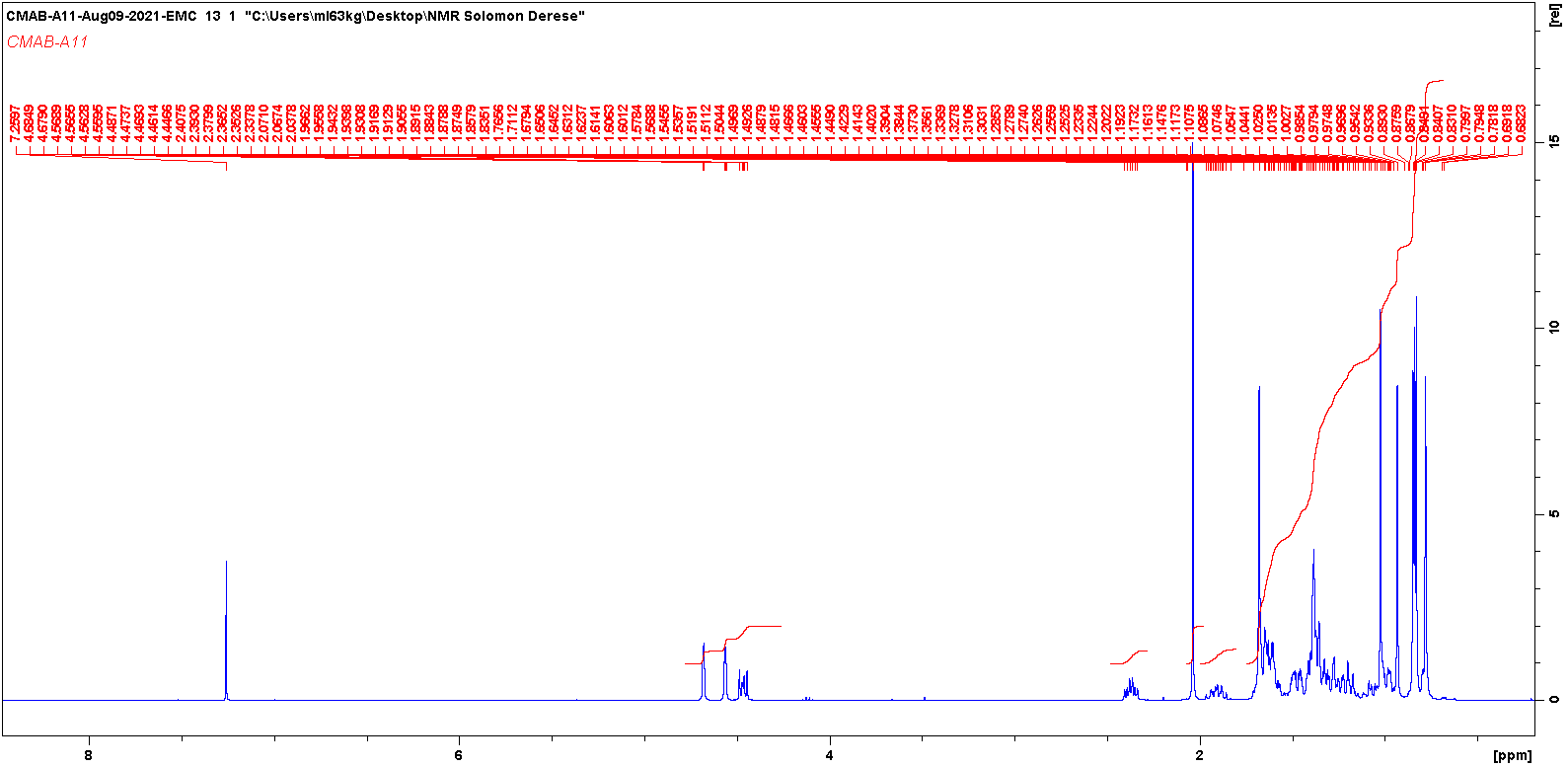
**

# **Figure 15 13C NMR spectrum of Lupeol acetate (3)**

**
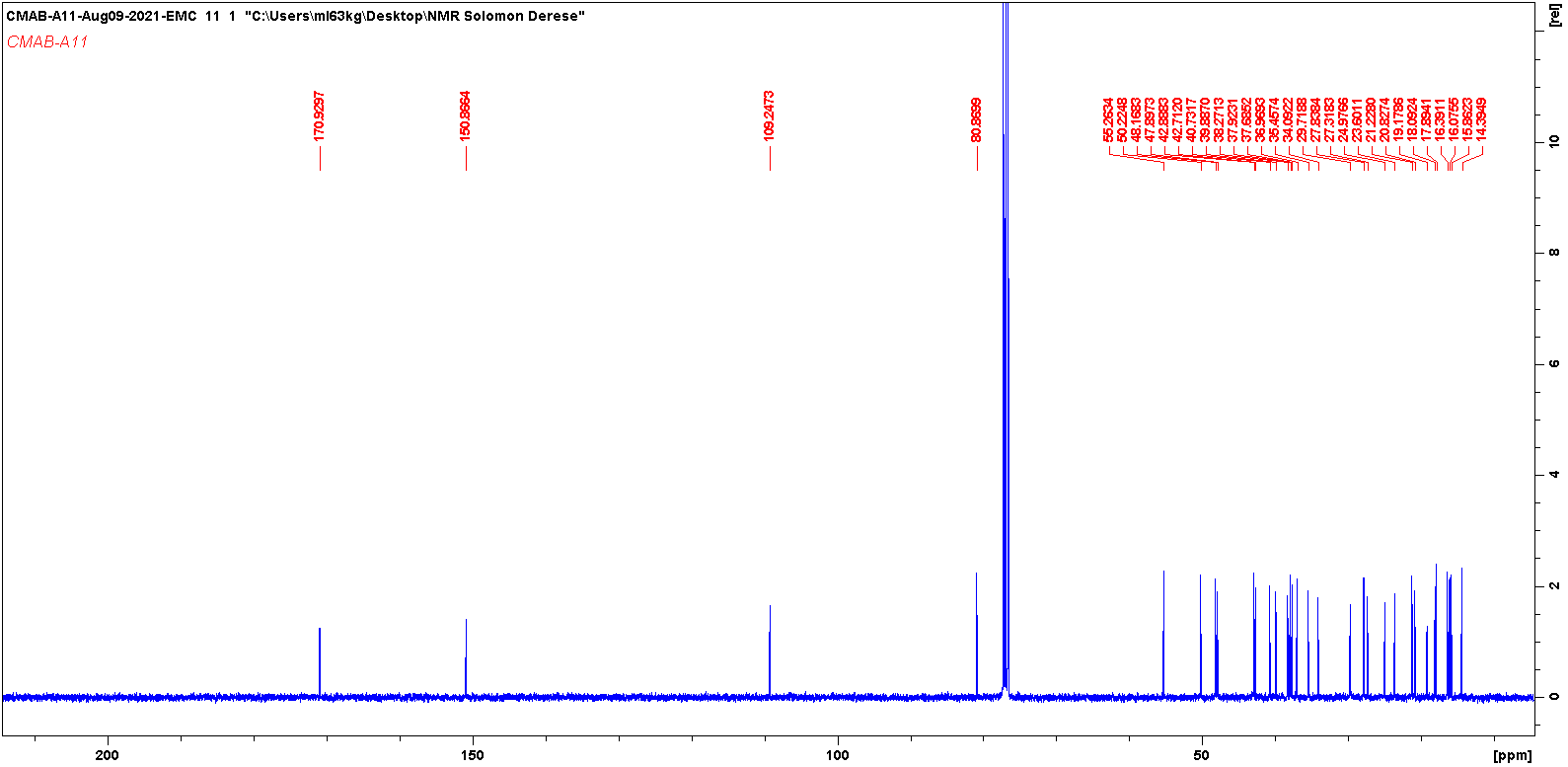
**

# **Figure 16 DEPT spectrum of Lupeol acetate (3)**

**
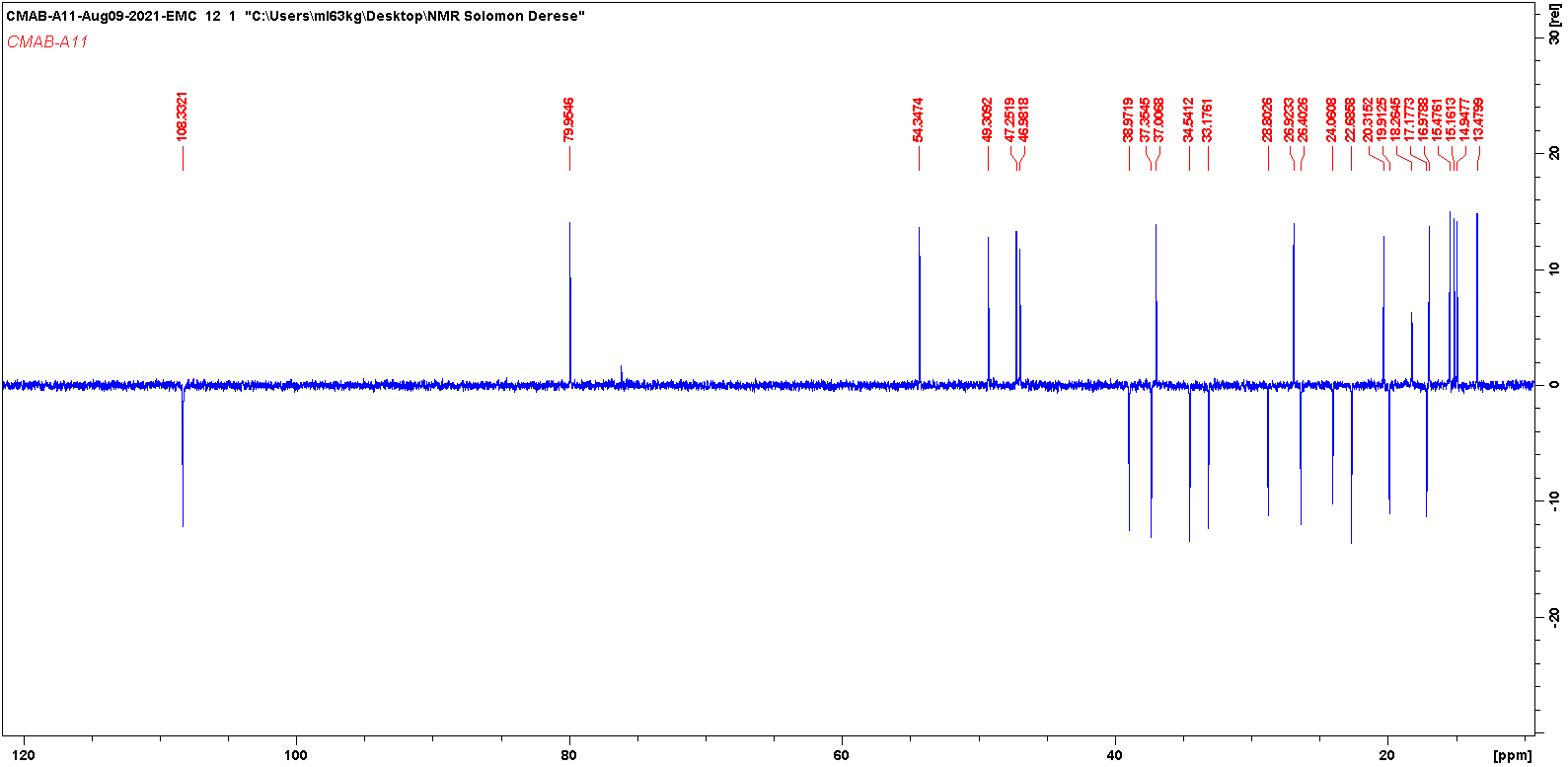
**

# **Figure 17 HSQCDEPT spectrum of Lupeol acetate (3)**

**
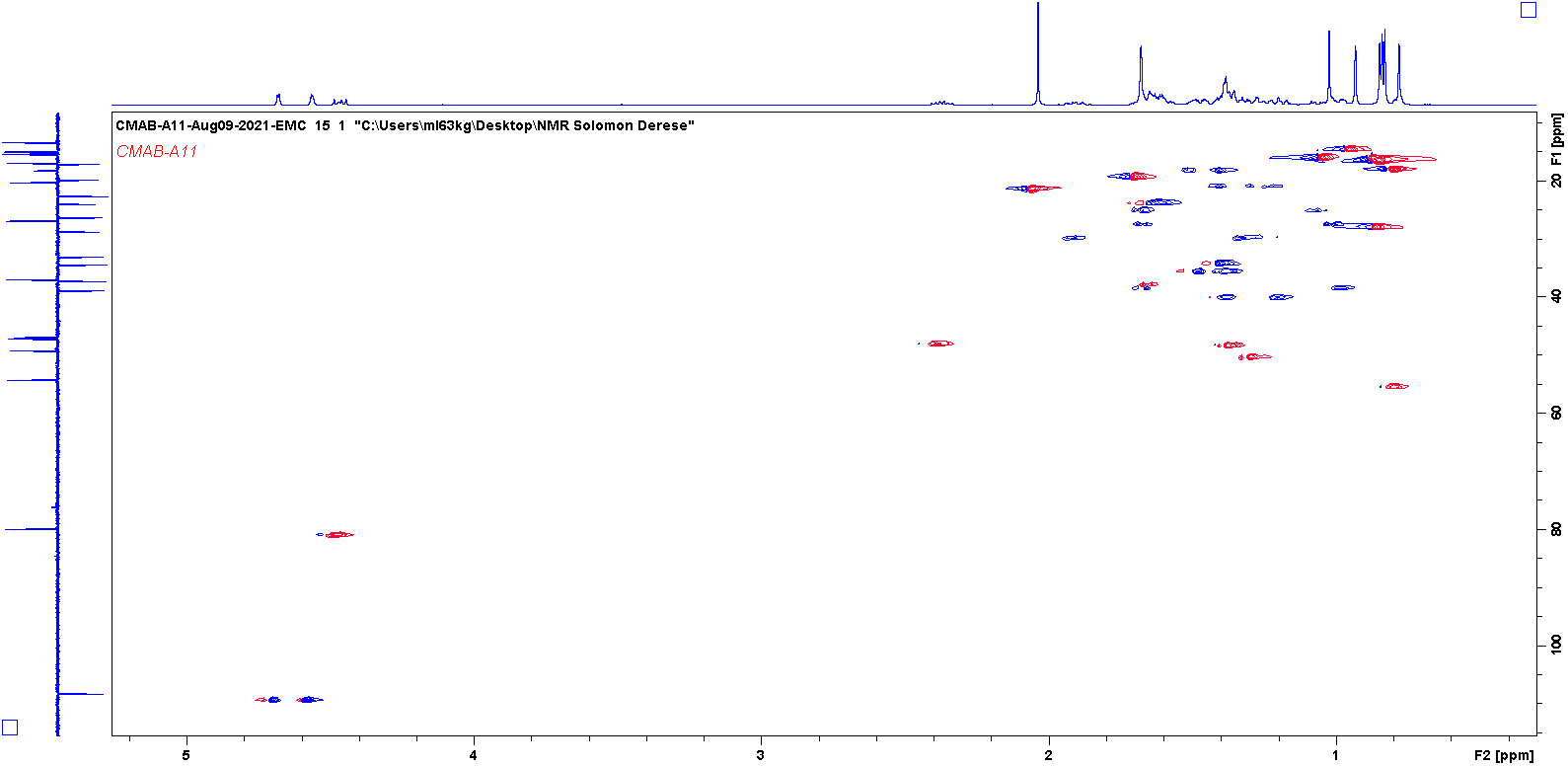
**

# **Figure 18 HMBC spectrum of Lupeol acetate (3)**

**
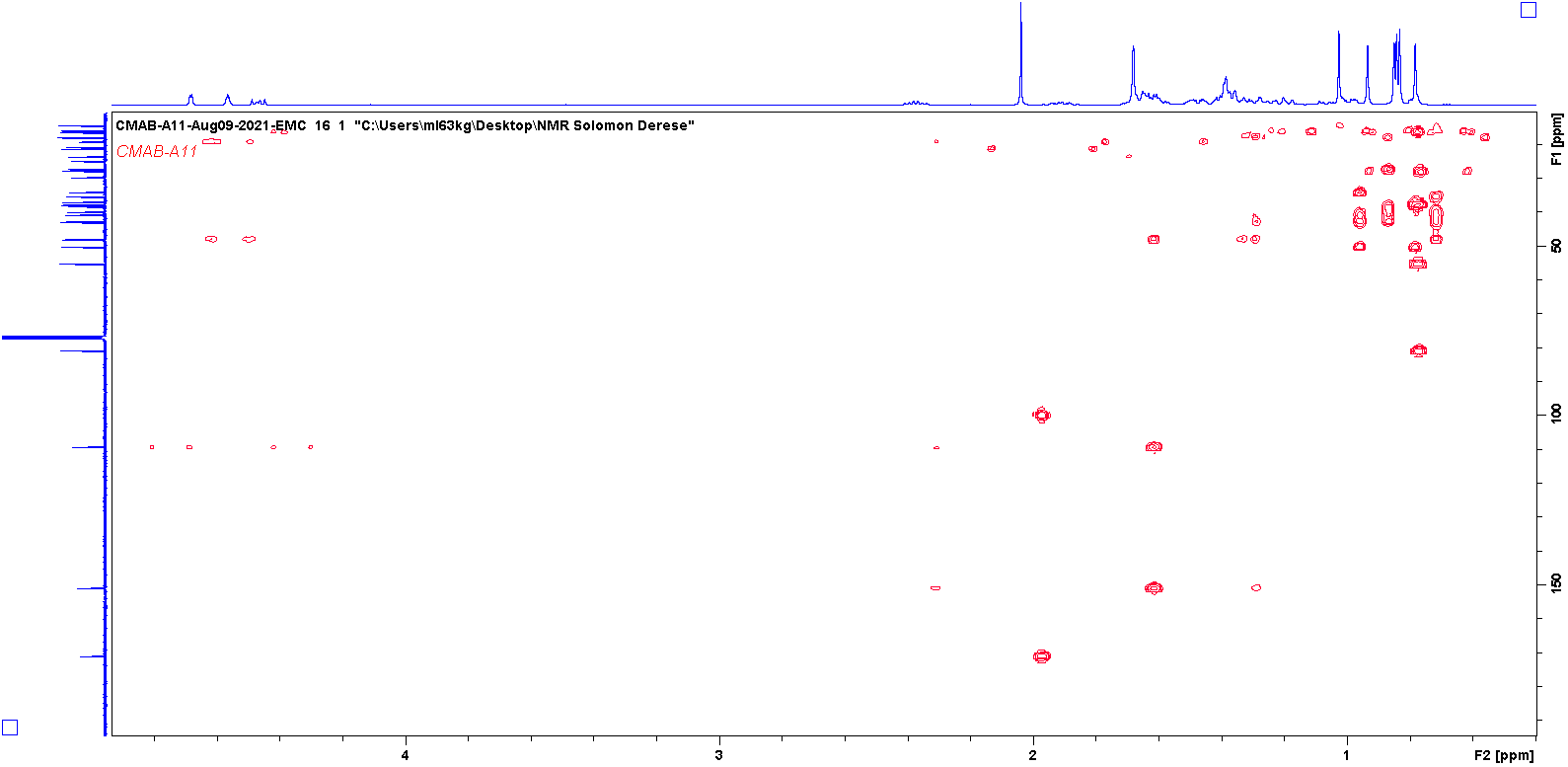
**

# **Figure 19 COSY spectrum of Lupeol acetate (3)**

**
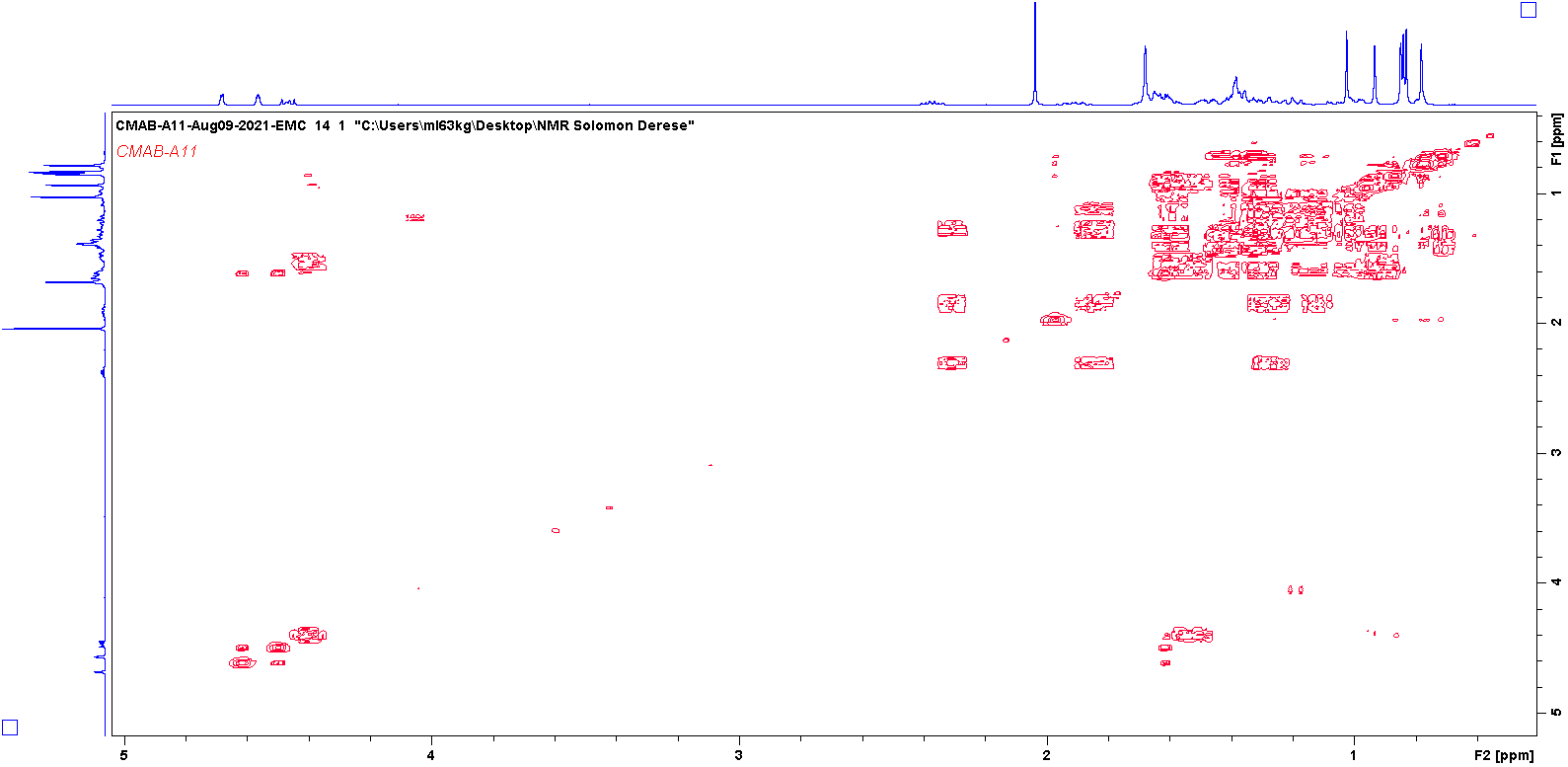
**

# **Figure 20 NOSEY spectrum of Lupeol acetate (3)**

**
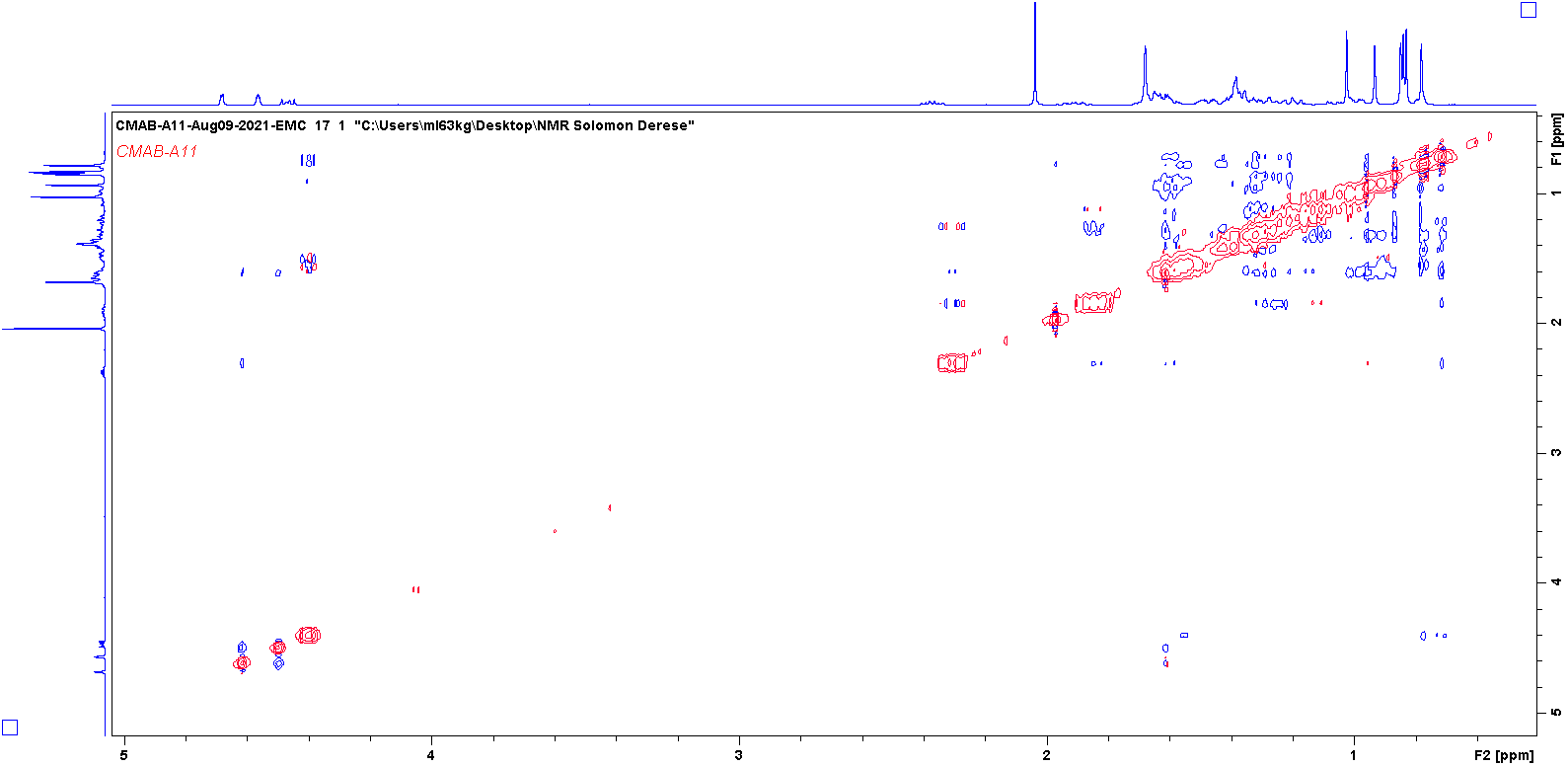
**

# **Figure 21 1H NMR spectrum of Betulin (4)**

**
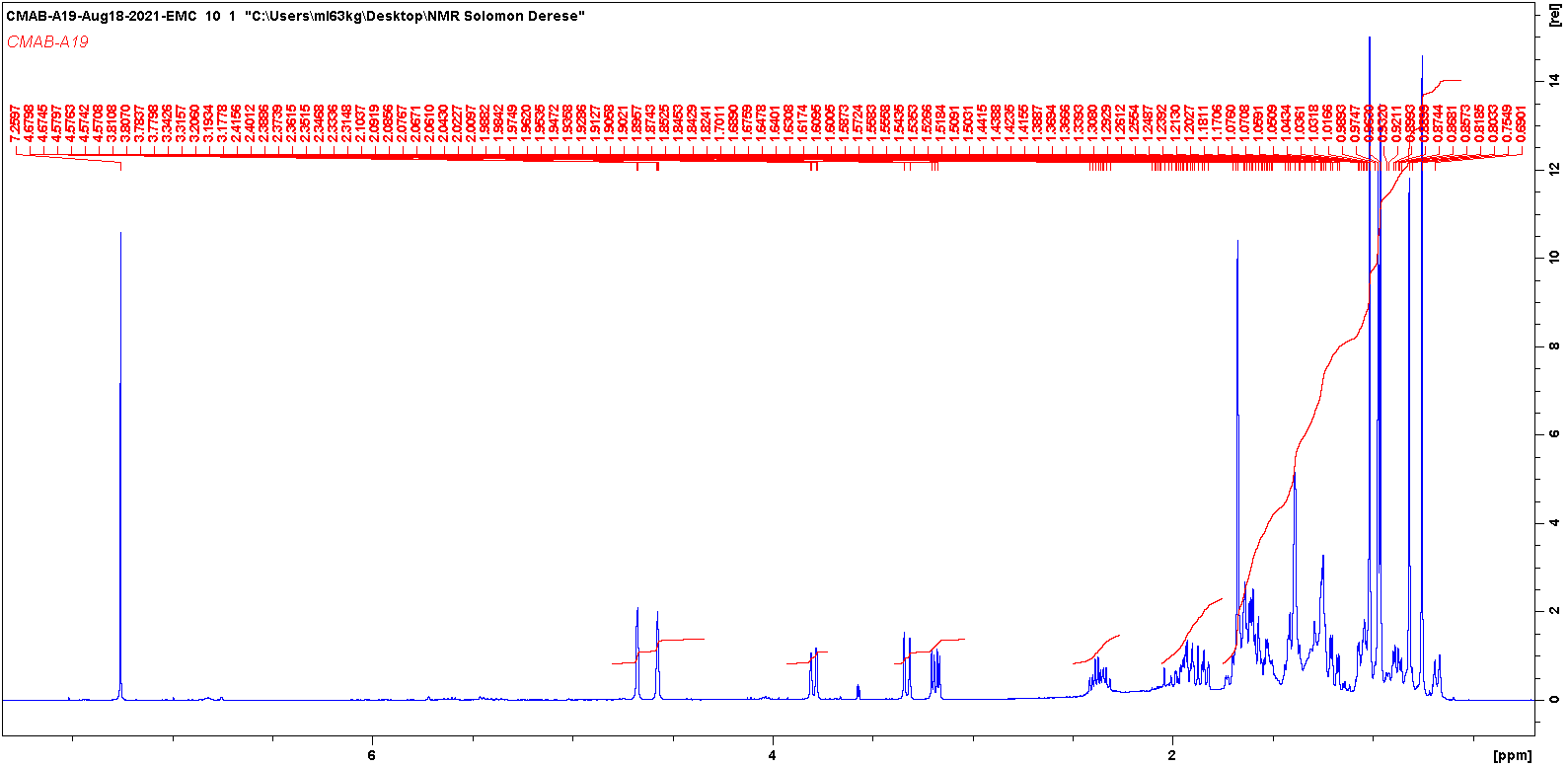
**

# **Figure 22 13C NMR spectrum of Betulin (4)**

**
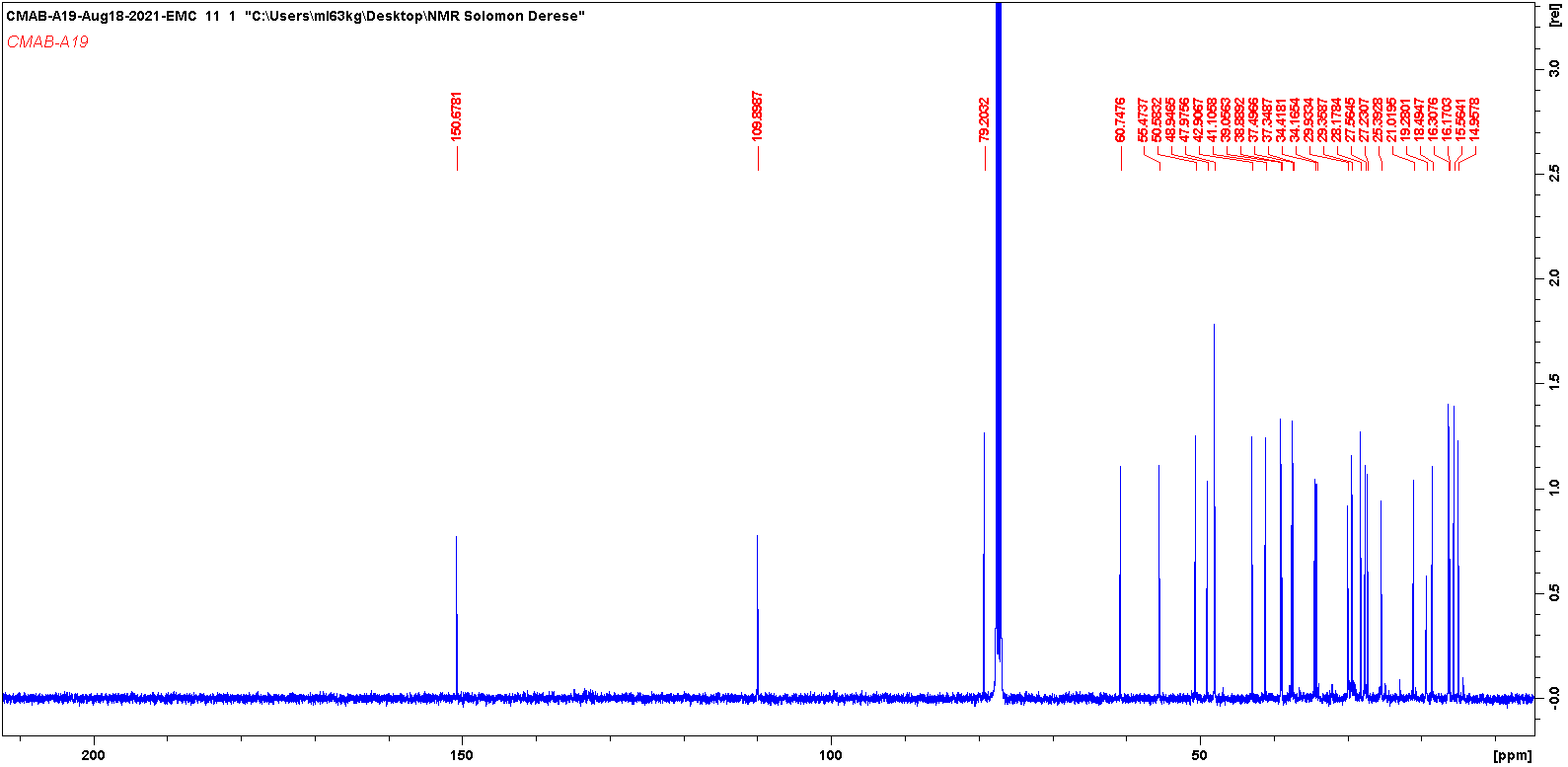
**

# **Figure 23 DEPT spectrum of Betulin (4)**

**
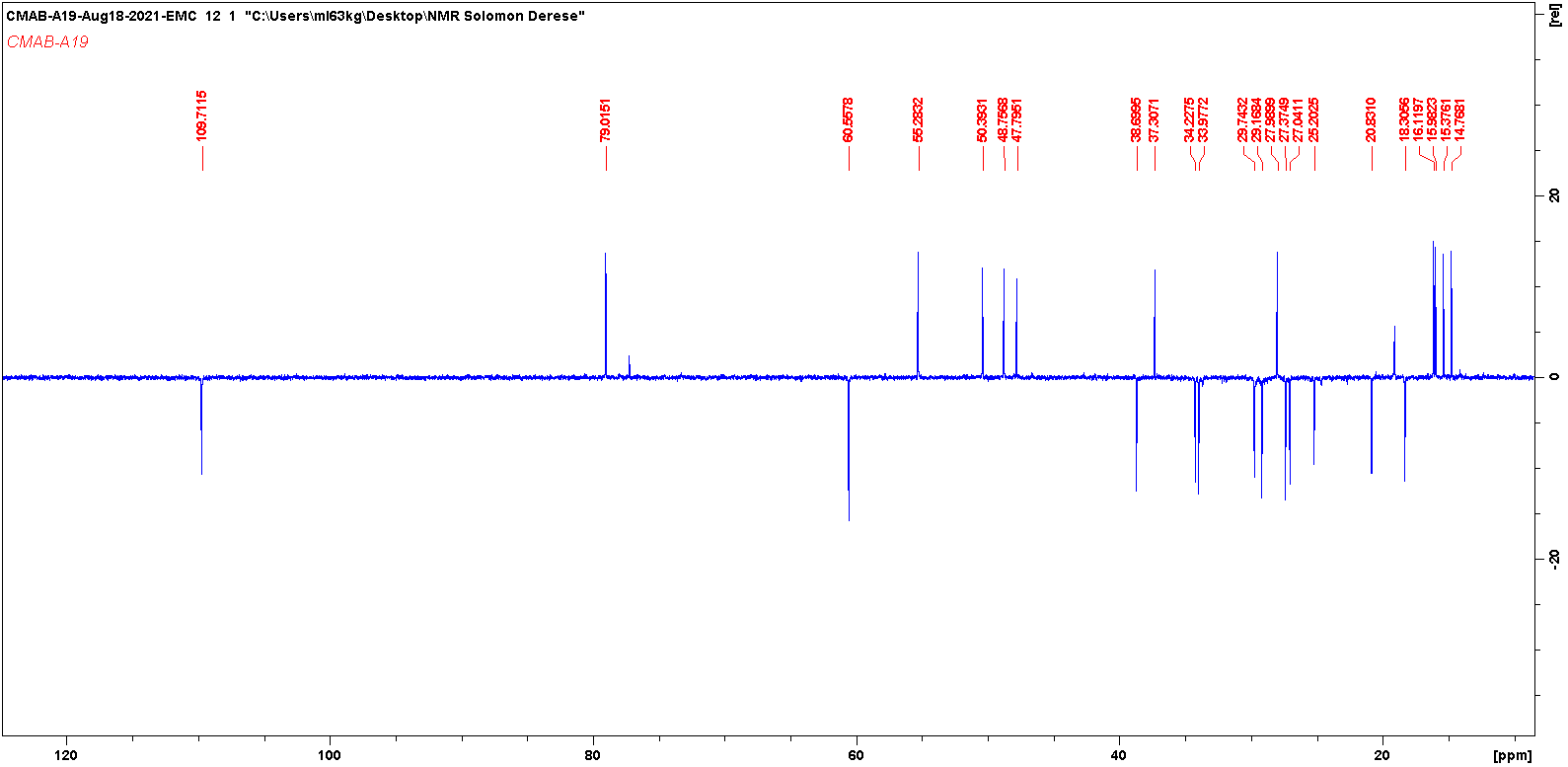
**

# **Figure 24 HSQCDEPT spectrum of Betulin (4)**

**
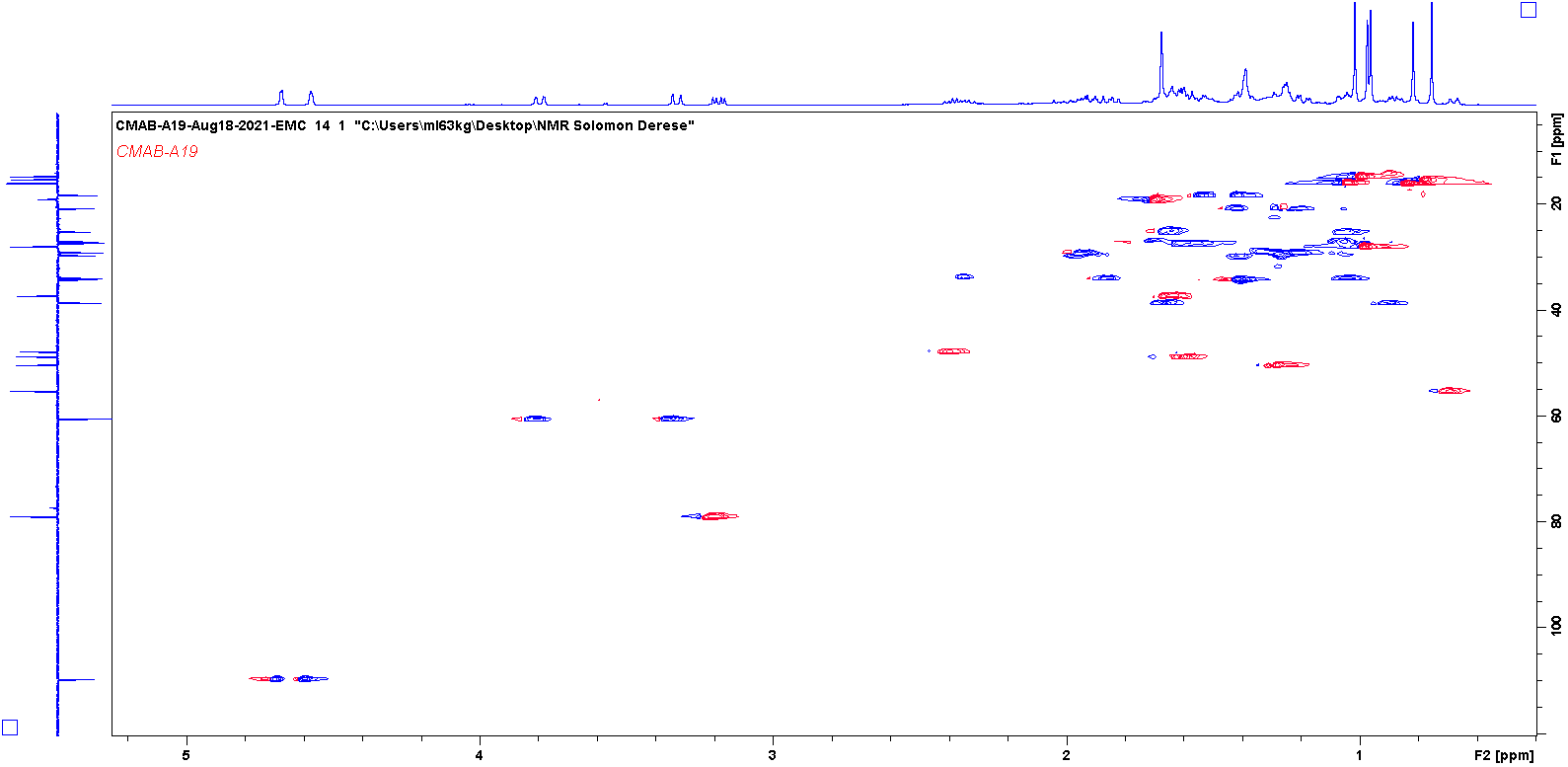
**

# **Figure 25 HMBC spectrum of Betulin (4)**

**
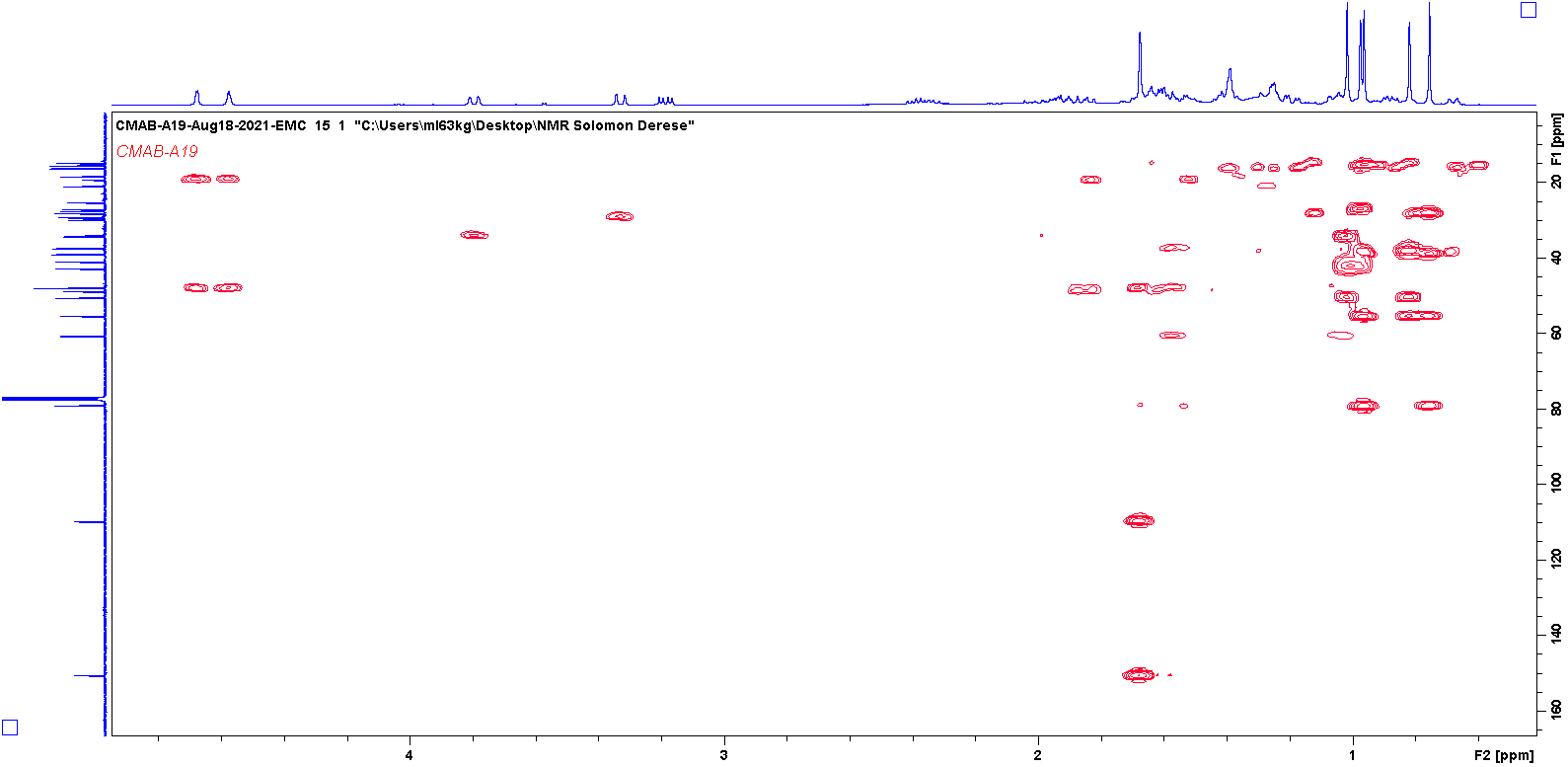
**

# **Figure 26 COSY spectrum of Betulin (4)**

**
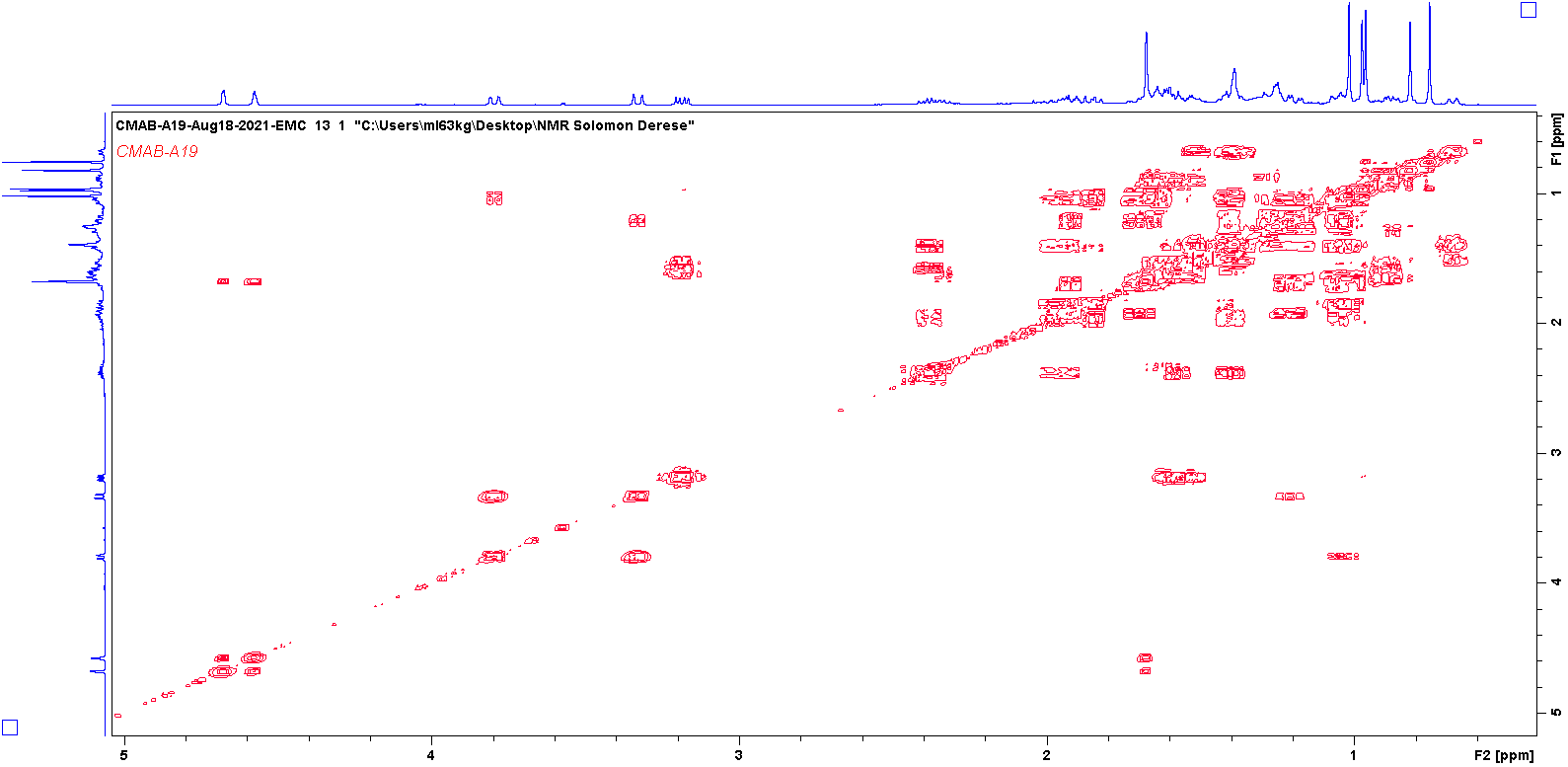
**

# **Figure 27 NOSEY spectrum of Betulin (4)**

**
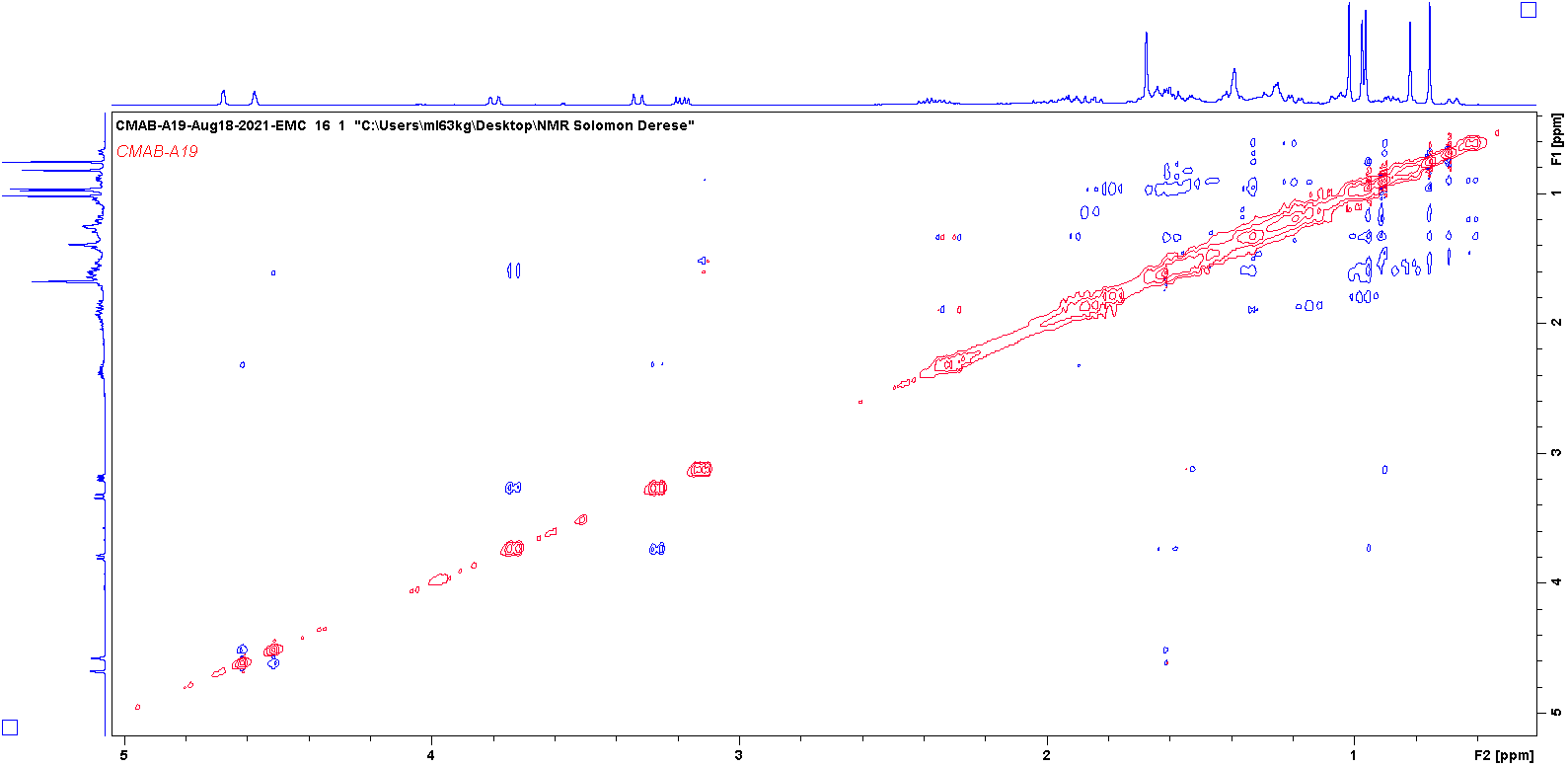
**

# **Figure 28 1H NMR spectrum of Lupeol (5)**

**
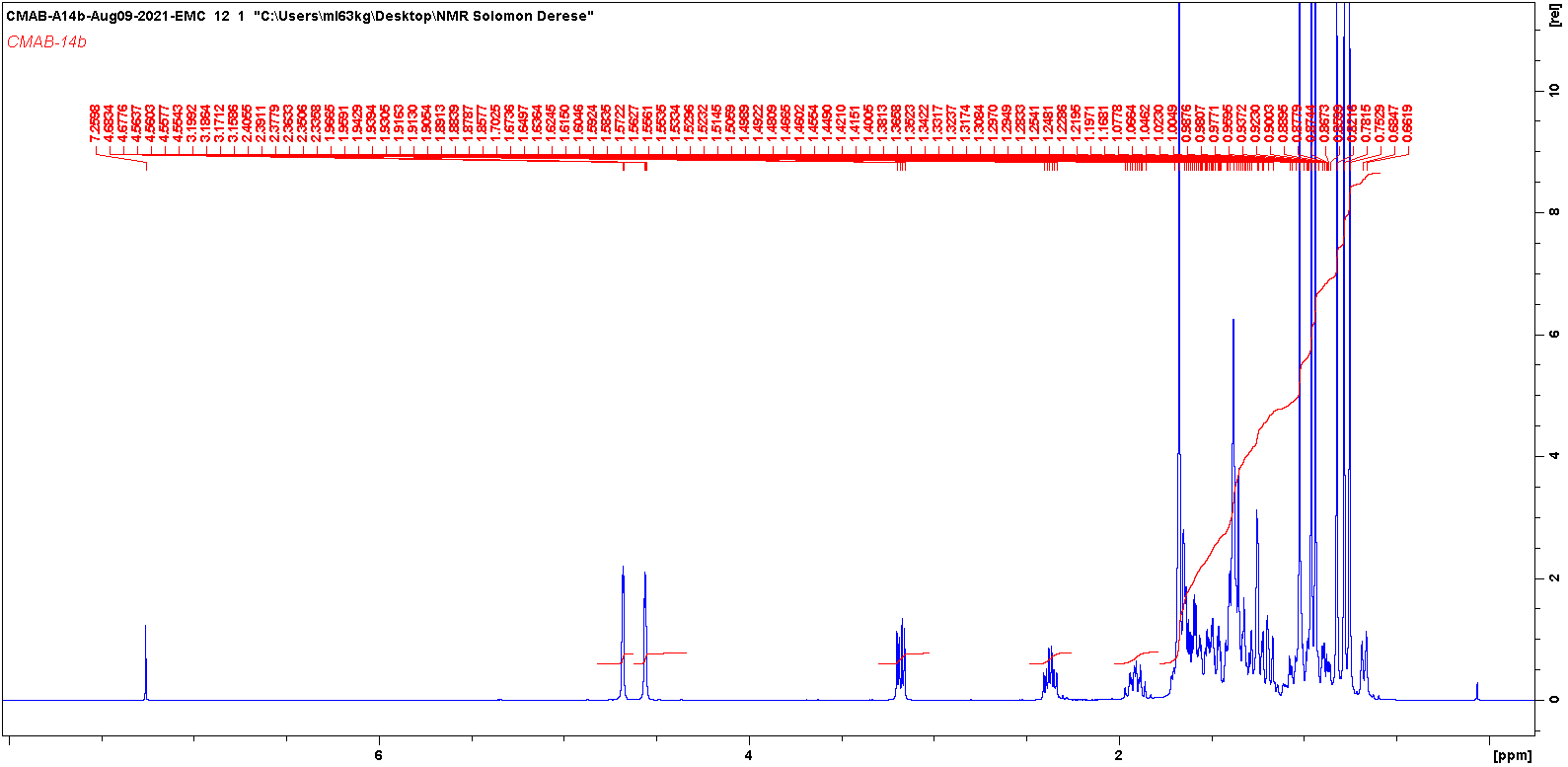
**

# **Figure 29 13C NMR spectrum of Lupeol (5)**

**
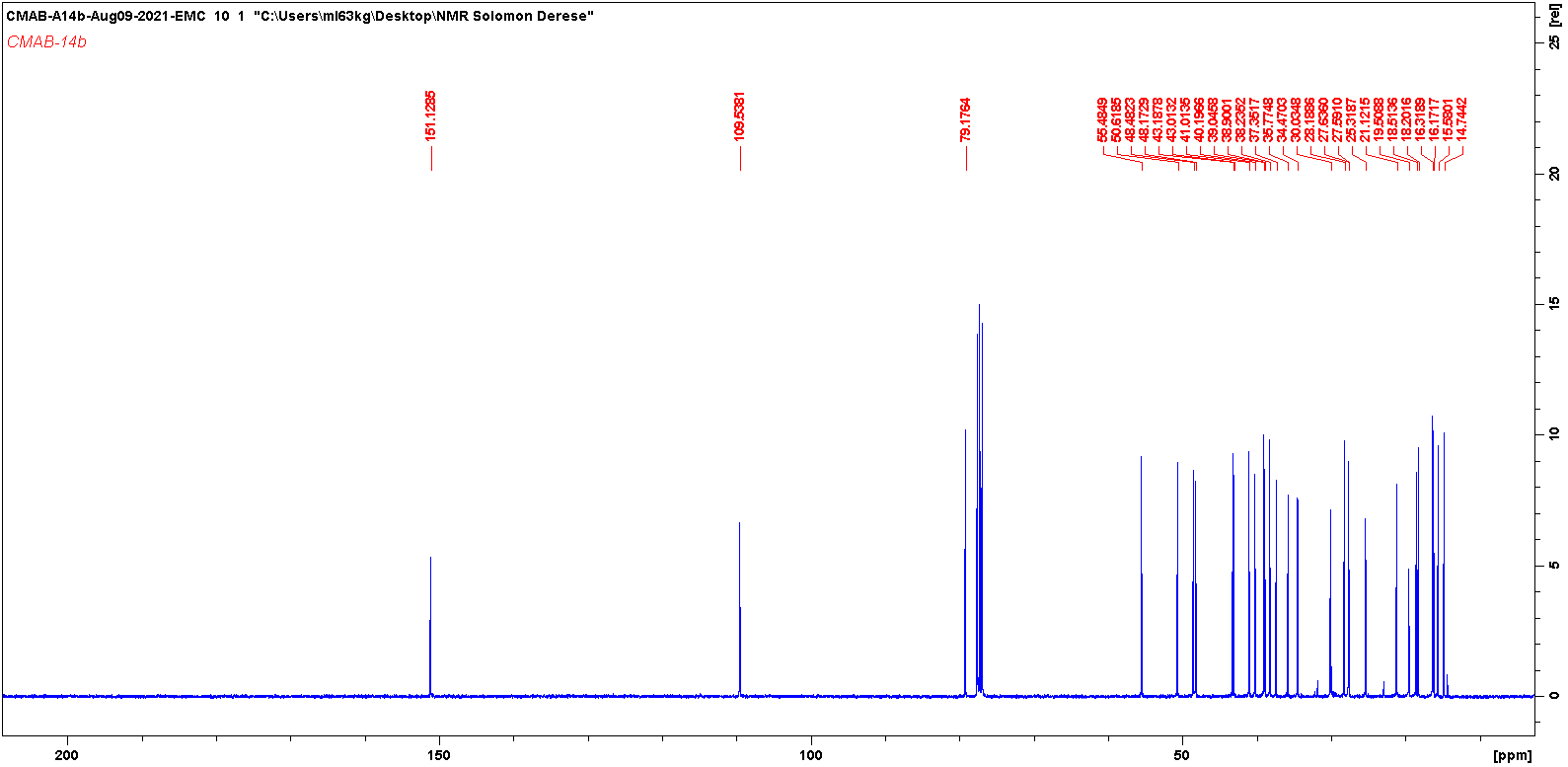
**

# **Figure 30 DEPT spectrum of Lupeol (5)**

**
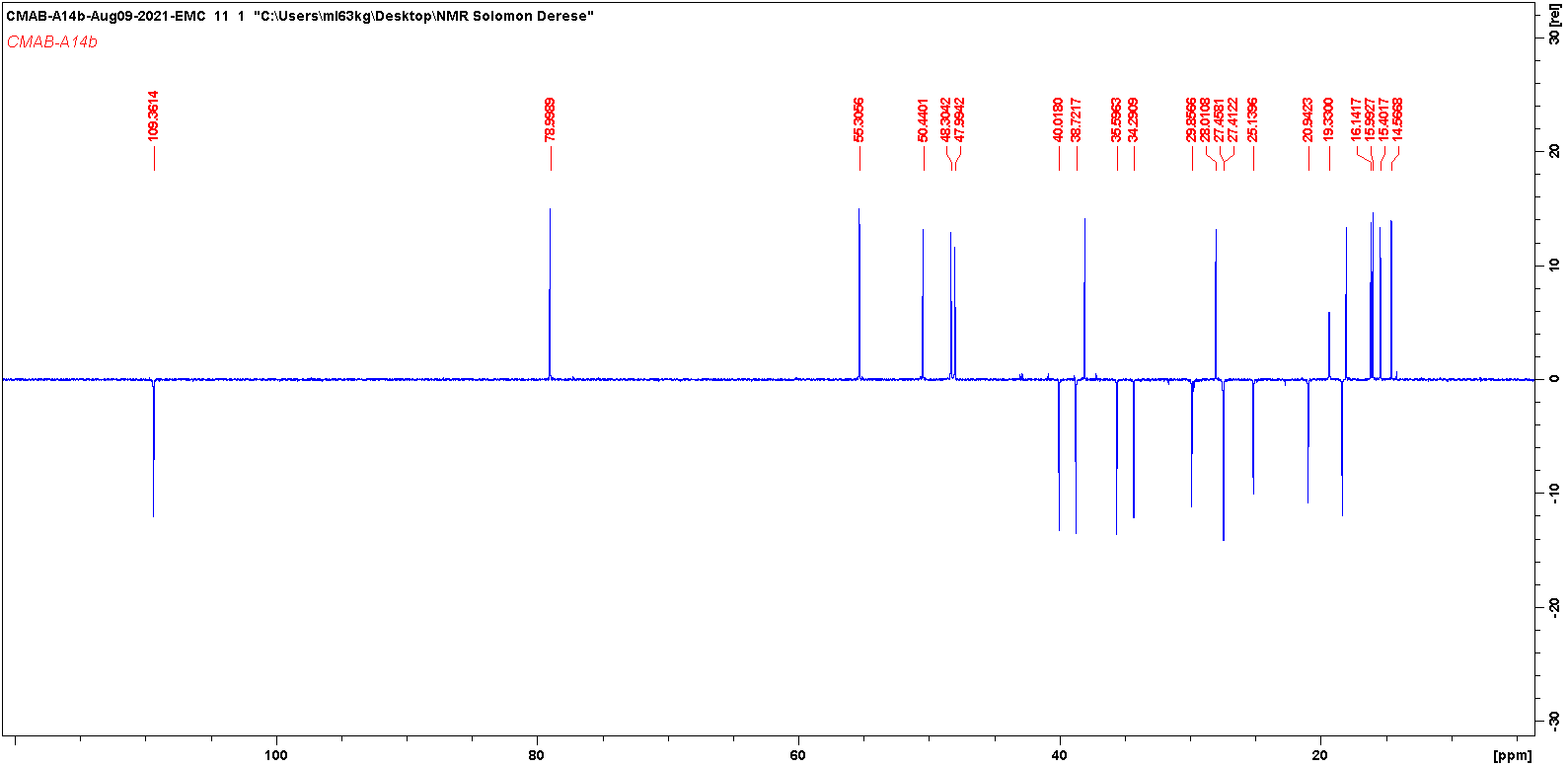
**

# **Figure 31 HSQCDEPT spectrum of Lupeol (5)**

**
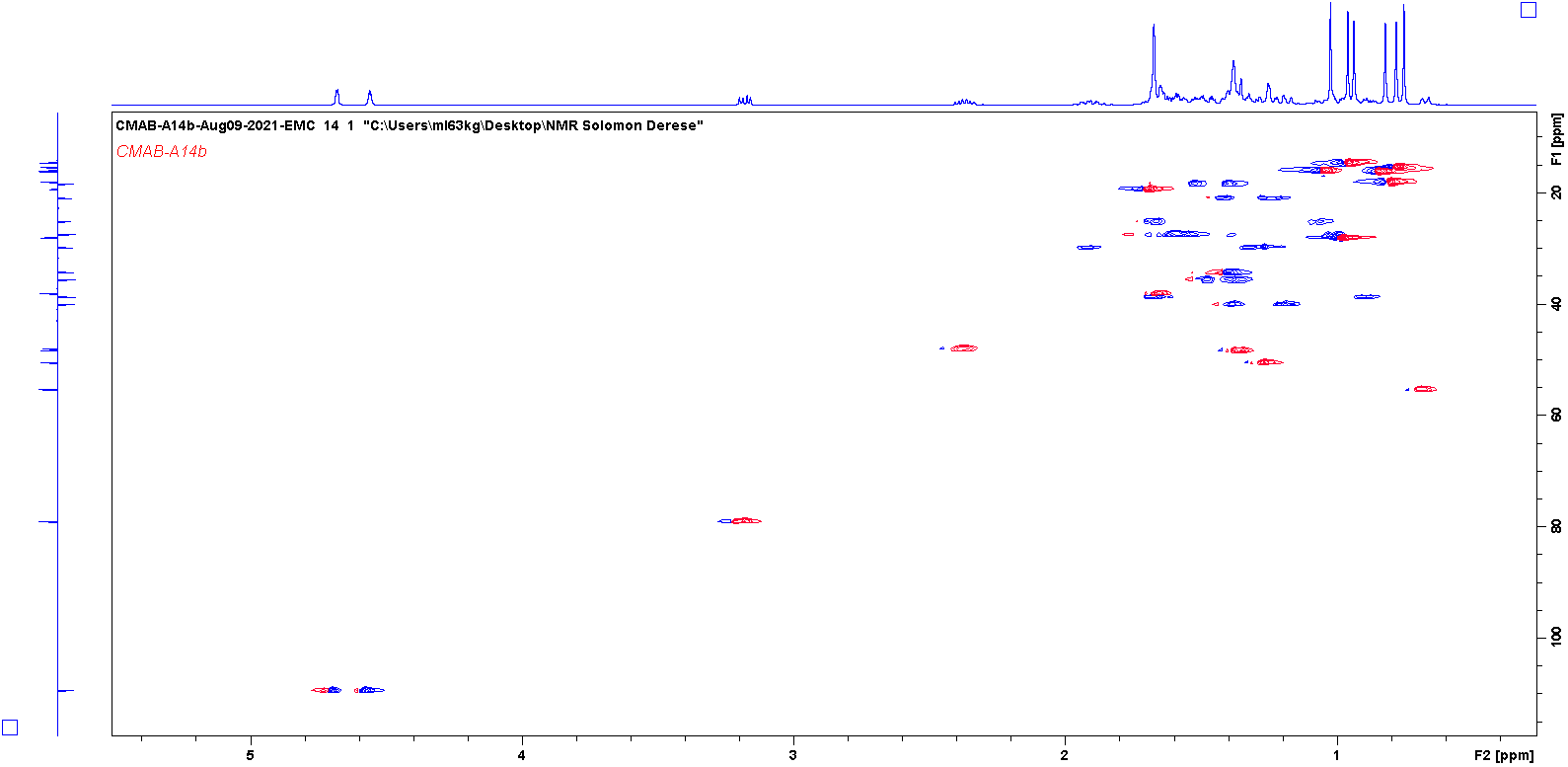
**

# **Figure 32 HMBC spectrum of Lupeol (5)**

**
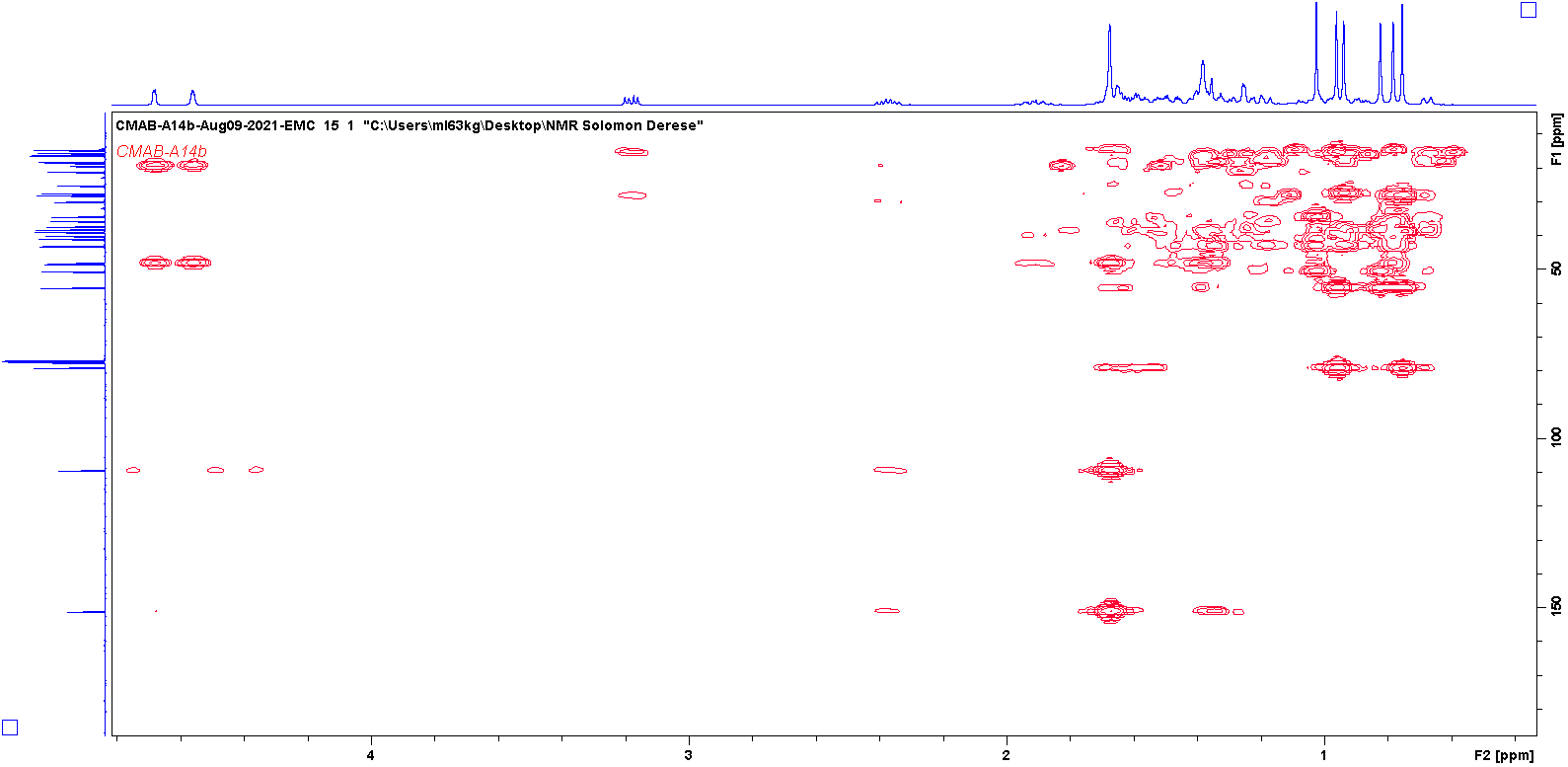
**

# **Figure 33 COSY spectrum of Lupeol (5)**

**
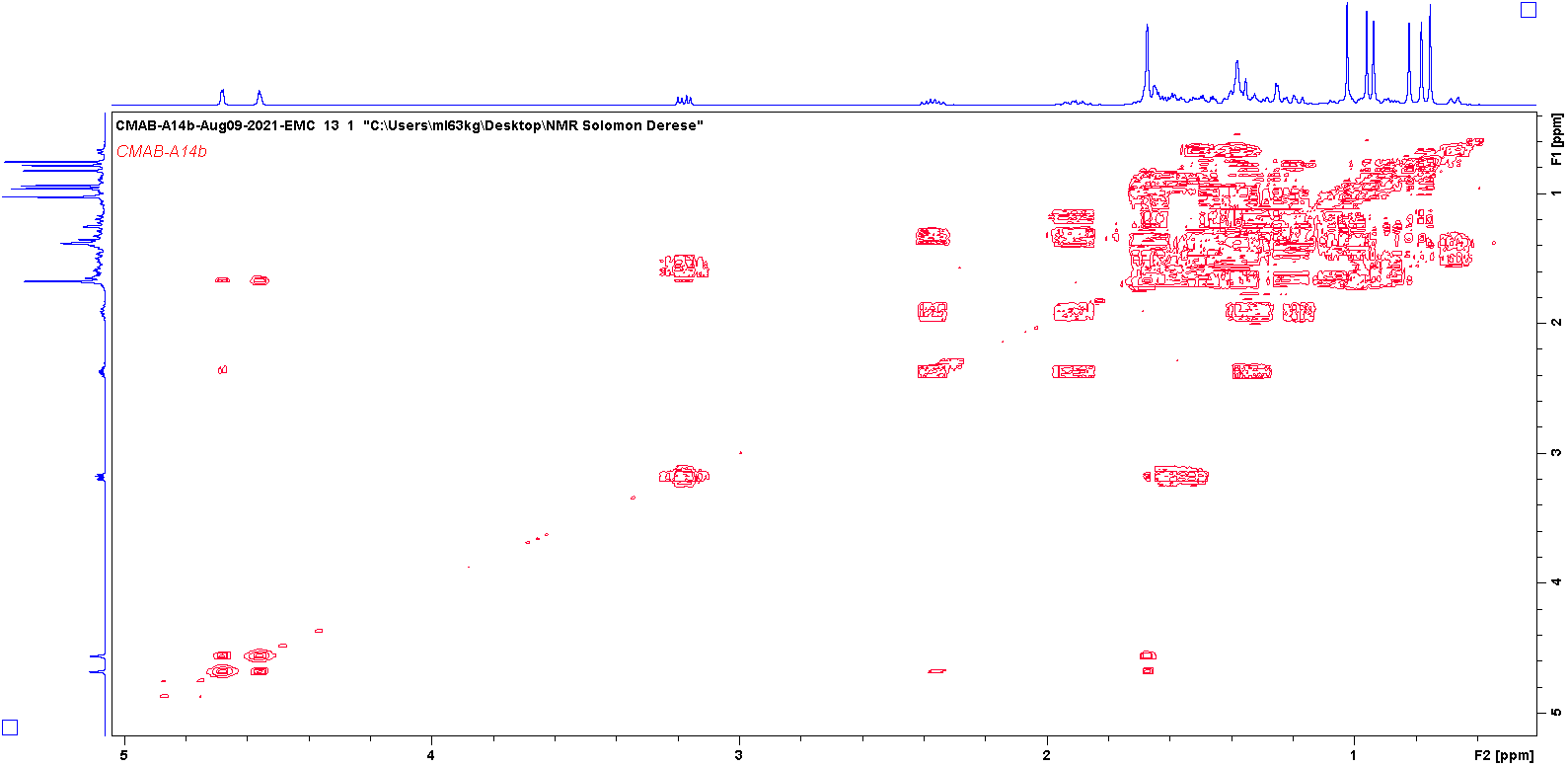
**

# **Figure 34 NOESY spectrum of Lupeol (5)**

**
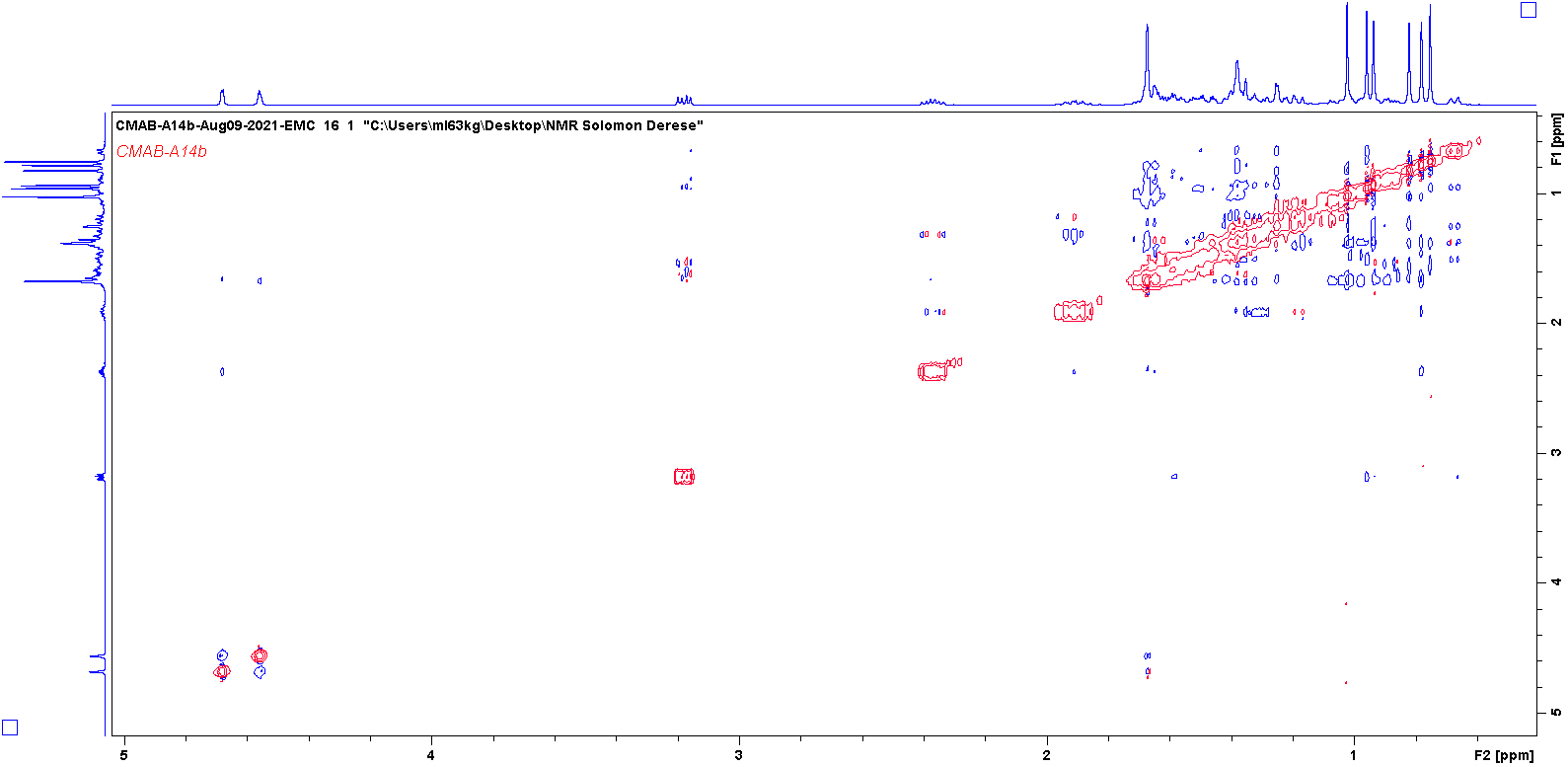
**

# **Figure 35 1H NMR Sitosterol (6) and stigmasterol (7)**

**
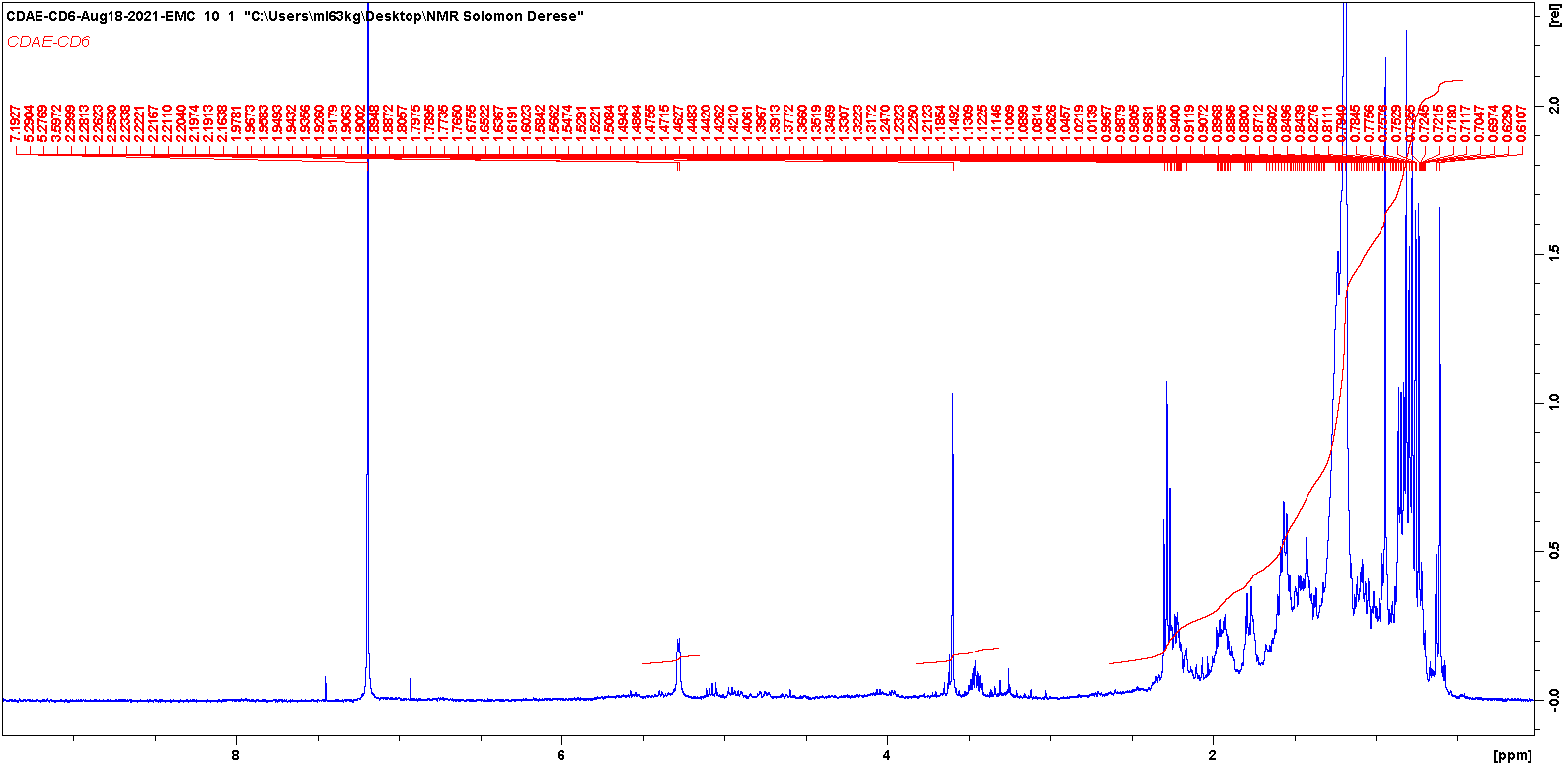
**
